# A Novel High-Throughput Screening Platform Identifies Itaconate Derivatives from Marine *Penicillium antarcticum* as Inhibitors of Mesenchymal Stem Cell Differentiation

**DOI:** 10.3390/md18040192

**Published:** 2020-04-05

**Authors:** Pietro Marchese, Nipun Mahajan, Enda O’Connell, Howard Fearnhead, Maria Tuohy, Janusz Krawczyk, Olivier P. Thomas, Frank Barry, Mary J. Murphy

**Affiliations:** 1 Regenerative Medicine Institute, School of Medicine, National University of Ireland Galway, H91TK33 Galway, Ireland; p.marchese1@nuigalway.ie (P.M.); frank.barry@nuigalway.ie (F.B.); 2 Marine Biodiscovery, School of Chemistry and Ryan Institute, National University of Ireland Galway, H91TK33 Galway, Ireland; npn.mahajan@gmail.com (N.M.); olivier.thomas@nuigalway.ie (O.P.T.); 3 Genomics and Screening Core, National Centre for Biomedical Engineering Science, National University of Ireland Galway, H91TK33 Galway, Ireland; enda.oconnell@nuigalway.ie; 4 Pharmacology and Therapeutics, School of Medicine, National University of Ireland Galway, H91TK33 Galway, Ireland; howard.fearnhead@nuigalway.ie; 5 Molecular Glycobiotechnology, School of Natural Sciences, National University of Ireland Galway, H91TK33 Galway, Ireland; maria.tuohy@nuigalway.ie; 6 Galway University Hospital, National University of Ireland Galway, School of Medicine, H91YR71 Galway, Ireland; janusz.krawczyk@nuigalway.ie

**Keywords:** marine fungi, high throughput screening, stem cell, regenerative medicine, drug discovery, *Penicillium antarcticum*

## Abstract

Worldwide diffused diseases such as osteoarthritis, atherosclerosis or chronic kidney disease are associated with a tissue calcification process which may involve unexpected local stem cell differentiation. Current pharmacological treatments for such musculoskeletal conditions are weakly effective, sometimes extremely expensive and often absent. The potential to develop new therapies is represented by the discovery of small molecules modulating resident progenitor cell differentiation to prevent aberrant tissue calcification. The marine environment is a rich reserve of compounds with pharmaceutical potential and many novel molecules are isolated from macro and microorganisms annually. The potential of small molecules synthetized by marine filamentous fungi to influence the osteogenic and chondrogenic differentiation of human mesenchymal stem/stromal cells (hMSCs) was investigated using a novel, high-throughput automated screening platform. Metabolites synthetized by the marine-derived fungus *Penicillium antarcticum* were evaluated on the platform. Itaconic acid derivatives were identified as inhibitors of calcium elaboration into the matrix of osteogenically differentiated hMSCs and also inhibited hMSC chondrogenic differentiation, highlighting their capacity to impair ectopic calcification. Bioactive small molecule discovery is critical to address ectopic tissue calcification and the use of biologically relevant assays to identify naturally occurring metabolites from marine sources represents a strategy that can contribute to this effort.

## 1. Introduction

Uncontrolled behaviour of progenitor/stem cells is involved in the progression of several degenerative diseases. For example, the inappropriate differentiation of these cells contributes to ectopic calcification in diffused pathologies such as vascular calcification in atherosclerosis or osteoarthritis [1,2] and is also associated with rare degenerative diseases such as fibrodysplasia ossificans progressiva or synovial osteochondromatosis [3,4]. A two to five times increased risk of vascular calcification is also observed in patients with chronic kidney disease, another widely diffused pathology with a mortality rate above 20% [5]. On the other hand, deficient bone generation is associated with degenerative diseases such as osteoporosis as well as rare heritable disorders like osteogenesis imperfecta [6,7].

Pharmacological treatments for defective bone conditions such as osteoporosis are currently available on the market as anti-resorptive, anabolic or dual-action agents [8,9]. However, pharmacological control of undesired calcification has not been addressed to date. Alternative approaches to treat musculoskeletal diseases focus on a regenerative medicine strategy, e.g., human mesenchymal stem or stromal cell (hMSC) transplantation for tissue regeneration. MSCs are multipotent cells able to differentiate along osteogenic and chondrogenic lineages when treated with appropriate growth factors and/or small molecules (SMs) in vitro. Bone morphogenetic proteins (BMPs) are important drivers of hMSC osteogenic differentiation [10] and have been used to enhance fracture healing through local administration [11]. SMs used to induce hMSCs osteogenic differentiation in vitro are ascorbic acid, dexamethasone and β-glycerophosphate [12]. Differentiation is achieved by the synergistic effects of enhanced proliferation induced by ascorbic acid, differentiation stimulated by dexamethasone and calcium mineralization promoted by β-glycerophosphate [13,14,15].

Although factors influencing MSC fate have been widely investigated and several compounds demonstrated bioactivity in vitro and in vivo, full pharmacological control of MSC differentiation has not been defined to date. Selective regulation of MSC differentiation by SMs represents a strategy to inhibit or improve tissue generation, with several advantages compared to the use of growth factors. Some SMs are more stable compared to protein, are generally non-immunogenic because of their small size, and can be delivered orally, thus avoiding repetitive painful injections [16,17]. Thanks to their ability to diffuse through the cell membrane, some SMs can also directly trigger intracellular targets without interacting with membrane receptors, thus inducing a quicker response, and can act where disease-associated decreases in cell receptors may be at play [18]. SMs are also able to perturb specific functions of single or multiple proteins in a reversible manner with temporal control [19]. Compared to recombinant proteins, SMs are easier to produce at scale and may, in some cases, be represented by inexpensive organic compounds [18]. Several SMs isolated from the marine environment are, at present, marketed for the treatment of cancer, pain, viral infections or hypertriglyceridemia, and many more are in clinical trial assessment [20]. In the field of musculoskeletal diseases, previous studies showed that fungal-derived statins were able to modulate MSCs differentiation ultimately leading to bone formation in vitro and in vivo [21] and evidence of pro-osteogenic potential was demonstrated from a variety of other marine organisms [22]. Considering the huge number of SMs isolated every year from the natural environment or chemically synthetized, the extensive screening of compounds to assess their bioactivity toward MSCs might lead to the discovery of strong and reliable pharmacological treatments to control cell fate. Such compounds would contribute to the development of new drugs for musculoskeletal diseases by preventing aberrant tissue calcification, stimulate the fate of implanted cells or improve the differentiation of endogenous stem cells.

Here, we present a high throughput screening (HTS) platform for the detection of molecules that can inhibit, promote or induce the osteogenic or chondrogenic differentiation of hMSCs. The miniaturized assays were developed to fit a 96-well plate format and procedures were optimized for compatibility with automated workstations. In order to minimize variability in the screening and obtain Z’ scores compatible with reliable hit detection, streamlined assay protocols with minimal plate manipulation were established. An initial comparison of differentiation performances of primary and hTERT MSCs was performed to define the best cell candidate for the platform. Osteogenic differentiation was measured as the cells’ ability to mineralize calcium, and results were normalized by the cell number obtained by counting nuclei using a high content imaging system. Chondrogenic differentiation was evaluated as the production of sulphated glycosaminoglycans (GAGs), normalized to cell number, measured as total DNA. Particularly promising for the biosynthesis of bioactive small molecules for these targets are filamentous fungi [21] and corals [22], therefore our efforts focused on the investigation of these organisms, applying an innovative high throughput approach. The presented HTS platform was implemented in the screening of various marine natural product libraries, including deep sea corals and sponges [23], as well as a small library of 12 pure natural products obtained from a strain of *Penicillium antarcticum* isolated in the North Atlantic Ocean. Here we report the bioactivity of the molecules isolated from *P. antarcticum* toward hMSC differentiation. Fungal metabolites were tested by addition to standard differentiation or to incomplete differentiation media to test their ability to inhibit, promote or induce hMSC differentiation.

## 2. Results

### 2.1. Osteogenic Differentiation Assay

#### 2.1.1. Differentiation Method Development

**Figure 1:**
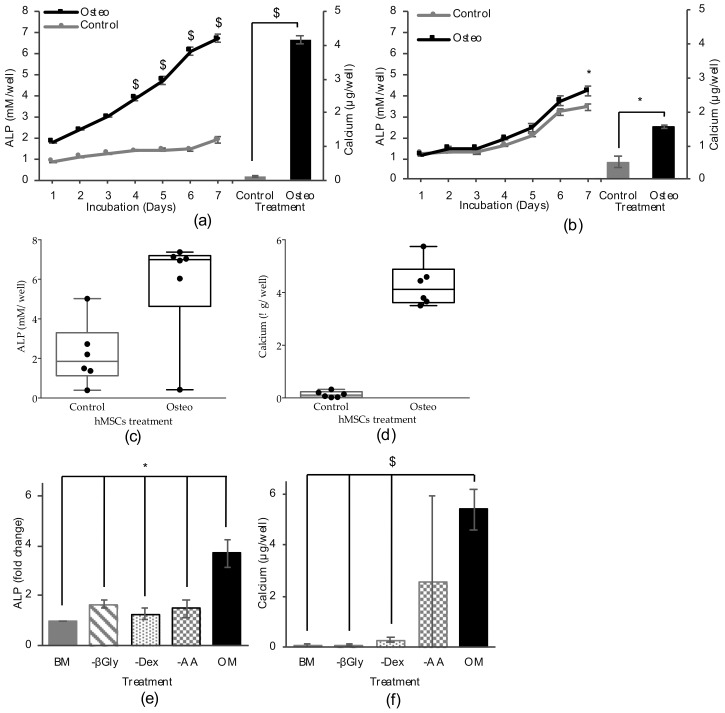
Development of the human mesenchymal stem/stromal cells’ (hMSCs) osteogenic differentiation assay. (**a**) Primary and (**b**) hTERT MSC osteogenic markers expression after treatment with osteogenic medium (Osteo) or growth medium (Control). Intracellular alkaline phosphatase (ALP) biosynthesis was measured (*N* = 1) every 24 hours for 7 days after treatment while calcium mineralization was measured after 10 days’ treatment. (**c**) Intracellular ALP expression and (**d**) calcium mineralization of primary hMSCs from six donors. Cells were treated with osteogenic medium (Osteo) or growth medium (Control) and incubated for 7 days to detect ALP expression and 10 days for calcium mineralization. The influence of single components of the osteogenic medium formulation on primary hMSC differentiation was evaluated measuring (**e**) intracellular ALP expression after 7 days, and (**f**) calcium mineralization after 10 days. The cells were treated with growth medium (BM), complete osteogenic medium (OM), OM lacking β-glycerophosphate (-βGly), OM lacking dexamethasone (-Dex) and OM lacking ascorbic acid 2-phosphate (-AA). Experiments were carried out on *N* = 1 donor for **a**, **b**; *N* = 6 for **c**, **d**, and *N* = 3 for **e**, **f**. Data are shown as the mean ± SD of 3 technical replicates, * indicates *p* ≤ 0.05 calculated using ANOVA one-way with Bonferroni post-test, $ indicates a Z’ factor > 0.5 calculated in comparison with the positive control.

Designing cellular assays for drug discovery first requires a careful choice of the cell type involved and the marker to detect bioactivity. Previously developed osteogenic assays for drug screenings involved primary hMSCs and measured the intracellular activity of alkaline phosphatase (ALP) using the enzyme-substrate reaction with *para*-nitrophenylphosphate as a differentiation marker [24,25]. Here, osteogenic differentiation of immortalized hTERT and primary hMSCs was evaluated using both intracellular ALP activity and calcium mineralization as differentiation markers. In addition to the selection of the optimal cell type, the reliability of HTS methods is strongly dependent on the assay length and number of steps required. In the osteogenic assay developed, no medium change was performed during the assay after treatment with osteogenic inducers. As shown in Figure 1a, primary hMSCs treated for differentiation as described above (DAG treatment) expressed increasing activity of ALP over the incubation period, reaching a five-fold difference compared to control cells at day seven. Primary hMSCs efficiently mineralized calcium after DAG treatment and were able to reach a Z’ factor > 0.5 ($) for both ALP expression and calcium mineralization. hTERT-MSCs showed lower marker expression for ALP activity and calcium mineralization (Figure 1b) not reaching required differentiation levels and showing a Z’ factor < 0.5 for both assays. Primary hMSCs were therefore selected for further assay development and metabolite screening.

To validate the DAG treatment method for osteogenic differentiation and identify the most reliable marker to be used in the screening, primary hMSCs obtained from six donors were treated for differentiation using the same procedure. Alkaline phosphatase activity after seven days differentiation was variable in the six hMSCs preparations (Figure 1c). DAG treatment induced increasing expression of ALP in five out of six donors compared to the untreated cells, while one donor did not show any difference in ALP levels between treated and untreated cells. For three of the donors used, differences in ALP activity between treated and untreated cells resulted in a Z’ factor > 0.5. In terms of mineralized calcium, all six donors showed increased calcium levels in the matrix of the treated cells compared to the untreated control (Figure 1d). Moreover, all donors were differentiated sufficiently to pass the Z’ factor threshold of 0.5, making this marker the most reliable for implementation in bioactivity screenings with a more uniform expression demonstrated in the various hMSC donors.

#### 2.1.2. Screening Conditions: Differentiation Medium and Marker Detection

**Figure 2:**
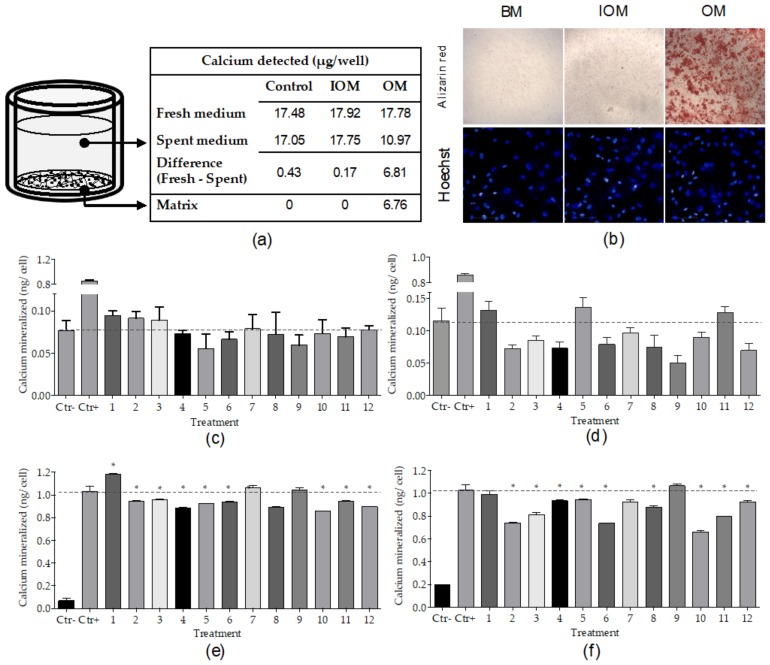
High-throughput osteogenic assay development and fungal metabolites screening. (**a**) Schematic representation of micro-well calcium detection using direct and non-direct methods. Primary hMSCs were cultured in Basic Medium (BM), Incomplete Osteogenic Medium (IOM—BM plus ascorbic acid and β-glycerophosphate) or Osteogenic Medium (OM—BM plus ascorbic acid, β-glycerophosphate and dexamethasone) and incubated for 10 days; media alone with no cells in wells were included as a control. After incubation, calcium was measured in the cell medium (spent medium), in the media used (fresh medium) and in the extracellular matrix (matrix) after treatment with 0.5M HCl. The matrix mineralized calcium, measured by an indirect method, was calculated by the difference between fresh medium and spent medium. (**b**) Primary hMSCs imaged after treatment and 10 days incubation in 96-well plates: matrix calcium was stained using Alizarin red S and cell nuclei stained using Hoechst 33342. The high-throughput osteogenic assay was implemented to screen 12 purified marine-derived metabolites. Primary hMSCs cultured in flat-bottom 96-well plates were treated with natural products and incubated for 10 days. The differentiation induction was tested by treating the cells with the natural products dissolved in IOM at 1 µM (**c**) or 10 µM (**d**). Positive control cells (Ctr+) were treated with OM, negative control cells (Ctr-) were treated with IOM. Promotion or inhibition of differentiation was tested by treating the cells with the natural products dissolved in OM at 1 µM (**e**) or 10 µM (**f**). Ctr+ cells were treated with OM, Ctr- cells were treated with IOM. Screening was carried out on experimental triplicates and presented as the mean ± SD, * indicates *p* ≤ 0.05 calculated using ANOVA one-way with Bonferroni post-test.

In vitro osteogenic differentiation of hMSCs is induced by the synergic activity of several compounds; for this reason, an accurate medium formulation is required to establish the experimental conditions that are suitable for the identification of new osteogenic inhibitors, promoters or inducers during screenings. The osteogenic activity of each individual component of the DAG mix was evaluated by treating three hMSC donors with an osteogenic medium (OM) lacking either dexamethasone, ascorbic acid or β-glycerophosphate in a comparison to differentiation induced by complete OM. Of the three donors tested, calcium mineralization was consistently affected by lack of dexamethasone or β-glycerophosphate, while the absence of ascorbic acid blocked the calcium mineralization of only two of the three donors tested (Figure 1e). A Z’ factor > 0.5 was obtained comparing cell differentiation after treatment with OM (positive control) to treatments lacking dexamethasone or β-glycerophosphate (negative controls), indicating the assay’s ability to detect potential osteogenic inducers in the absence of a single compound in the hMSC osteogenic assay. ALP activity at day seven showed significantly lower levels after treatment with each of the three incomplete osteogenic media, compared to OM-treated cells (Figure 1f), as evidenced by the Z’ factor > 0.5 obtained. To test differentiation induction by novel compounds, OM-lacking dexamethasone (Incomplete Osteogenic Medium (IOM)) was chosen as the formulation to be used during the screening. The promotion or inhibition of differentiation was screened in complete OM. Cells differentiated using the protocol described above were stained using Alizarin red S. Treatment with BM, and IOM showed no significant calcium mineralization compared to the OM-treated cells (Figure 2b).

#### 2.1.3. High Throughput Osteogenic Assay

Platforms for high throughput drug discovery use robotic and automated liquid handling systems designed for multi-well plates and assays. Here, we performed a streamlined protocol involving only two steps for a 96-well plate set up: cell seeding and treatment after overnight incubation, followed by 10 days of differentiation without intermediate media changes. From the perspective of further streamlining of the protocol and to preserve cells for quantification, an indirect method to detect mineralized calcium was established. This method is based on the quantification of calcium depleted from the medium by the hMSC after differentiation rather than the quantification of the matrix mineralized calcium performed in the standard direct method.

In order to validate our procedure, the standard direct method to quantify mineralized calcium was compared to the indirect method developed. Detected calcium levels were found to be comparable between the two procedures for the assessment of hMSC osteogenesis (Figure 2a). The screening platform was able to detect marker expression indirectly, allowing further analysis of the cell layer and thus enabling a streamlined differentiation protocol optimal for the implementation of high-throughput drug discovery studies. A Z’ factor of 0.82 was obtained comparing IOM (negative control) and OM (positive control), demonstrating the assay’s ability to identify new osteogenic differentiation inducers, as a substitute activity of that exerted by dexamethasone. Additionally, a Z’ factor of 0.76 was obtained by comparing BM and OM, demonstrating the assay’s ability to detect osteogenic promoters or inhibitors, in comparison to the current standard in vitro differentiation treatment.

### 2.2. Chondrogenic Assay

#### 2.2.1. Differentiation Method Development

**Figure 3:**
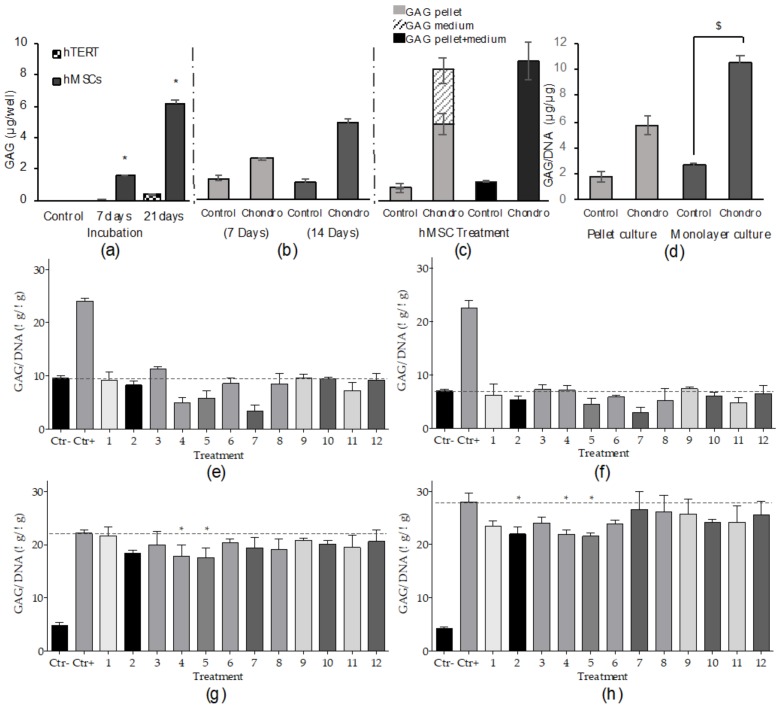
High throughput chondrogenic assay development and fungal metabolites screening. (**a**) Sulphated glycosaminoglycans (GAG) production by hMSCs after 7 and 21 days differentiation of primary and hTERT MSCs stimulated by 10ng/mL TGF-β3. Control cells were fed with Incomplete Chondrogenic Medium (ICM) without addition of growth factor. (**b**) Production of GAG by primary hMSCs at 7 and 14 days after treatment with 10ng/mL TGF-β3. Control cells were treated with ICM. (**c**) GAG elaborated by primary hMSCs in pellet format after 14 days chondrogenic differentiation. Pellet GAG is represented in grey while the medium GAG level is the striped area. GAG level quantified from pellet and medium digested together is shown in black. (**d**) Comparison of pellet and monolayer culture assessed by GAG produced after 14 days differentiation. Data represent GAG quantified in cell aggregates and medium. Results are presented as the mean ± SD of 3 technical replicates from 1 donor (**a**, **b**, **c**) or 3 donors (**d**). $ indicates a Z’ factor > 0.5. For screening, primary hMSCs cultured in flat-bottom 96-well plates were treated with marine compounds for 14 days. Differentiation induction was tested by treating the cells with the compounds dissolved in ICM at 1 µM (**e**) or 10 µM (**f**). Positive control cells (Ctr+) were treated with CCM, negative control cells (Ctr-) were treated with ICM. Differentiation promotion or inhibition was tested by treating cells with compounds dissolved in CCM at 1 µM (**g**) or 10 µM (**h**). Ctr+ cells, treated with CCM; Ctr- cells treated with ICM. Results are presented as the mean ± SD of 3 technical replicates, * indicates *p* ≤ 0.05 calculated using ANOVA one-way with Bonferroni post-test.

The initial assessment of pro-chondrogenic activity focused on selection of the optimal cell to use for drug screening. The differentiation of primary and hTERT hMSCs was compared. MSCs require both stimulation with differentiation factors and high-density culture to undergo chondrogenic differentiation. Under these conditions, MSCs synthetize extracellular matrix (ECM) components such as the proteoglycan aggrecan with extensive GAG side chains [26,27]. As the elaboration of the chondrogenic matrix takes several weeks, an initial comparison of short- and long-term differentiation was performed to evaluate chondrogenesis in the two cell types. Primary hMSCs showed significant differentiation in terms of the GAG contained in the pellets compared to the hTERT-MSCs at 7 and 21 days after treatment (Figure 3a). After 7 days differentiation, a significant increase in GAG biosynthesis was observed in primary hMSCs as compared to untreated controls, while hTERT-MSCs did not show any significant GAG production. Primary hMSC aggregate GAG content increased further after 21 days differentiation, while hTERT-MSC GAG remained low. As such, primary hMSCs were selected as the optimal cell type to be used in the subsequent optimization and screening of metabolites.

Despite the significant differentiation levels obtained, the detected marker level was not sufficient to reach the Z’ factor threshold of 0.5. In order to increase ECM biosynthesis to enable the achievement of a positive score, while maintaining a reasonable timeframe for the HTS platform, differentiation was extended from seven to 14 days. As shown in Figure 3b, a significant GAG increase was recorded after 7 days differentiation with a two-fold increase observed after 14 days. Moreover, the marker detection was performed on both the cellular aggregates and medium. Further optimization based on GAG detection from pooled aggregates and medium resulted in a 40% increase over that achieved using the pellet alone (Figure 3c).

#### 2.2.2. High-Throughput Chondrogenic Assay

A streamlined method to perform chondrogenic high-throughput screening with automated instruments was further optimized. Due to their shape, size and free movement in the medium, the potential loss of cell pellets through aspiration during robotic manipulations such as media changes was an issue. Moreover, the centrifugation step required to create condensed cell aggregates was time consuming, particularly in the context of the multiple plates required. To overcome this issue, a high-density monolayer culture system, not involving centrifugation, was compared to the standard pellet culture differentiation. The culture feeding system was also changed from the standard 50% media replacement to the addition of medium throughout the differentiation period. As shown in Figure 3d, both culture methods allowed the significant differentiation of the aggregates treated with chondrogenic medium. The use of the high-density monolayer culture generated a higher GAG/DNA ratio and resulted in a Z’ factor > 0.5, unlike the pellet culture. This method was therefore implemented for the screening of metabolites for chondrogenic differentiation on the platform.

### 2.3. Fungal Metabolites Isolation and Structure Elucidation

To investigate the pharmaceutical potential of Irish marine-derived filamentous fungi, a single strain isolated from the ascidian *Aplidium pallidum* (Verrill, 1871) was identified and grown for chemical extraction. Macro-morphological observation of the isolated colony showed dark brown-green mycelium and a pale reverse. Heavy conidiation was observed, along with the absence of pigments and the presence of exudates. Microscopical observation confirmed the presence of conidiophores typical of the genus *Penicillium*. Molecular identification based on the similarity to GenBank reference sequences for marker genes ITS, β-tubulin and calmodulin confirmed the isolated strain as *Penicillium antarcticum* [28].

**Figure 4:**
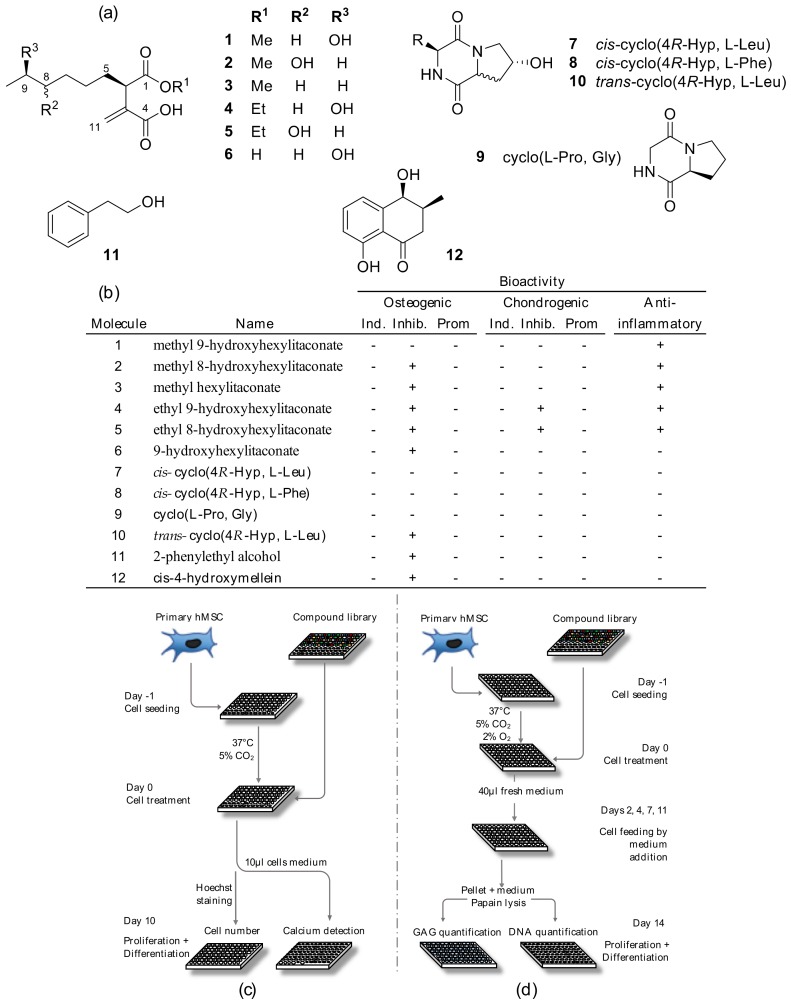
Marine fungal compounds structure, bioactivity summary and HTS platform schematic representation. (**a**) chemical structure of metabolites isolated from *Penicillium antarcticum*. (**b**) table summarizing the detected bioactivity of tested molecules: induction (Ind.), Inhibition (Inhib.) and promotion (Prom.). Compounds were considered positive (+) if bioactivity was detected at both concentrations tested, 1 and 10 µM. (**c**) Automated high-throughput osteogenic assay: primary hMSCs suspended in basic medium were seeded in flat-bottom 96-well plates (12 × 10^3^ per well), incubated overnight to let the cells settle and then treated with either test compounds or controls. After 10 days incubation, medium in each well was assayed for calcium quantification while the cell layer was stained using Hoechst 33342 for nuclei counting using the Operetta high content imaging system. (**d**) Automated high-throughput chondrogenic assay: primary hMSCs suspended in incomplete chondrogenic medium were seeded in flat-bottom 96-well plates (50 × 10^3^ per well), incubated overnight to obtain a high-density monolayer culture, and then treated with compounds or controls. GAG quantification was carried out after papain digestion using a DMMB assay while the DNA content quantified after staining with Hoechst.

Chemical investigation of the extract obtained from this strain led to the isolation of three new metabolites: ethyl 8-hydroxyhexylitaconate (**5**), methyl 8-hydroxyhexylitaconate (**2**) and ethyl 9-hydroxyhexylitaconate (**4**) along with nine known compounds: three hexylitaconic acid derivatives **1, 3** and **6** [29,30,31], four diketopiperazines **7**–**10** [32,33], 2-phenylethyl alcohol **11** and one isocoumarin **12** [34] (Figure 4a).

Compound **5** was isolated as a white powder and its molecular formula was established as C_13_H_22_O_5_ by (+)-HRESIMS with a sodium adduct at m/z 281.1360 [M + Na]^+^ indicating three indices of hydrogen deficiency. An inspection of the ^1^H NMR spectrum acquired in DMSO-d_6_ confirmed the presence of two methyl groups at δ_H_ 0.83 (t, J = 7.0 Hz, H_3_-10) and 1.14 (t, J = 7.0 Hz, H_3_-Et), one oxygenated methylene at δ_H_ 4.05 (q, J = 7.0 Hz, H_2_-Et) and two exomethylene olefinic protons at δ_H_ 5.71 (d, J = 2.0 Hz, H-11b) and 6.20 (d, J = 2.0 Hz, H-11a). These signals were characteristic of a hexylitaconic acid derivative. An oxygenated methine with signals at δ_H_ 3.28 (br s, H-8) and δ_C_ 70.7 (C-8) was different from the previously known compounds **1** and **6**. Key H_3_-10/H_2_-9/H-8 COSY correlations located the hydroxyl group at C-8. Because the chiral center C-2 was located next to a chromophore we did assign the absolute configuration at this center using electronic circular dichroism. The calculation of the ECD spectra for both epimers at C-8 having a S configuration at C-2 were superimposable and shown in the Supplementary Materials (Figure S10) with a unique positive Cotton effect at 230nm. Because the experimental spectrum of **5** exhibited a unique and negative Cotton effect at the same wavelength, we deduced a R configuration at C-2. However, the Mosher analysis was not conclusive for the chiral center at C-8 due to the low amount of material available and led to a rapid decomposition of the starting material. To the best of our knowledge, this is the first report of a hexylitaconic acid derivative with an alcohol at the position C-8.

Compound **2** had a molecular formula of C_12_H_20_O_5_ with the sodium adduct at *m/z* 267.1209 [M + Na]^+^. The ^1^H NMR spectrum of **2** was very similar to the spectrum of compound **5,** except for the absence of the signals corresponding to the ester ethyl group. Instead, new signals at δ_H_ 3.57 (s, H_3_-Me) and δ_C_ 51.5 (C-Me) revealed the presence of a methyl ester group. Compound **2** was, therefore, the methyl analogue of the ethyl ester **5** and the second example of a hexylitaconic acid with an alcohol at C-8.

Compound **4** was isolated as a white amorphous powder and showed the same molecular formula C_13_H_22_O_5_ as compound **5,** with a [M + Na]^+^ adduct at *m/z* 281.1361 in (+)-HRESIMS. The main differences between the ^1^H NMR spectra of **4** and **5** were in the oxygenated methine with signals at δ_H_ 3.53 (m, H-9) and δ_C_ 65.6 (C-9) present in the spectrum of **4**. The position of the hydroxyl group at C-9 instead of C-8 for **5** was confirmed by key H-10/H-9 COSY correlation. Compound **4** is, therefore, the ethyl analogue of the known methyl ester **1.** Even if the configuration at C-9 remains to be confirmed, we propose that both compounds **4** and **1** share the same configuration at this position as they should be biosynthetically related. Because **1** possessed the same rotatory power as the known compound reported in the literature, they should also the same R absolute configuration, especially at C-2. Together with the assessment of the 2R configuration for the new compound **5**, we assume that all compounds in this series have the same absolute configuration at this position due to biosynthetic homogeneity.

### 2.4. Fungal Metabolites Bioactivity Screenings

Once the optimal conditions for hMSC differentiation were set and the high-throughput assays developed, 12 marine fungal-derived pure metabolites were tested using the platform (Figure 4c,d). A first screening to evaluate the molecules cytotoxicity was performed by testing them at concentrations of 1 and 10 µM (method in the Supplementary Materials). Cells, recorded 72 hours after treatment, did not show loss of viability (Figure S1). Potential inducers of hMSC osteogenic differentiation were evaluated as a substitute of dexamethasone in the hMSC differentiation treatment, while differentiation promoters or inhibitors were tested by adding the compounds to the complete osteogenic medium (OM). Following the same principle, chondrogenic inducers were tested as a substitute of TGFβ-3 in the differentiation treatment, while promoters or inhibitors were tested in complete chondrogenic medium. Compounds dissolved in DMSO (final concentration 0.1% per well) were diluted in the appropriate medium and tested at two concentrations, 1 and 10 µM, in triplicate. To detect metabolites with osteogenic induction properties, the calcium mineralized after treatment was compared to the differentiation level of negative control cells (Figure 2c,d). Cells treated with 1 or 10 µM compounds did not show any significant positive variation in calcium mineralization compared to untreated cells. To identify compounds that promoted or inhibited differentiation, the level of mineralized calcium after treatment was compared to the positive control marker expression (Figure 2e,f). Cells treated with 1 µM compounds in the medium showed one molecule with the capacity to increase mineralized calcium per cell and eight molecules that inhibited calcium mineralization. The 10 µM treatment showed only inhibition of cell differentiation, with nine metabolites active in reducing calcium mineralization after treatment. To detect chondrogenic inducers, GAG production after treatment was compared to the differentiation level obtained by negative control treated cells (Figure 3e,f). Cells treated with compounds at 1 or 10 µM did not show any significant difference in extracellular matrix production compared to the negative control. To identify compounds promoting or inhibiting differentiation, the level of synthetized GAG after treatment was compared to the positive control marker expression. Two compounds were bioactive when tested at 1 µM (Figure 3g) and three at 10 µM (Figure 3h), significantly decreasing the level of GAG produced by the cells after treatment. For both osteogenic and chondrogenic screenings, the cell number was quantified in order to normalize the differentiation detected; data are reported in Supplementary Tables S1 and S2. The fungal compounds anti-inflammatory potential was also evaluated (method in Supplementary Materials) by treating LPS-activated THP1 macrophages with 1 µM fungal compounds. A medium level of the pro-inflammatory cytokine TNF-α was measured 6 h after treatment, showing decreased levels when the macrophages were treated with itaconic acid derivatives (compounds **1**, **2**, **3**, **4** and **5**, Figure S2).

The integration of results from all the bioactivities tested enabled the detection of the metabolites influencing MSCs’ ectopic calcification through differentiation to both lineages (Figure 4b). The itaconic esters (compounds **2**, **3**, **4** and **5**) as well as hexylitaconic acid (compound **6**), showed a significant inhibition of hMSCs osteogenic differentiation when tested at both 1 and 10 µM. Of these, the ethyl ester derivatives of itaconic acid (**4** and **5**) also showed the capacity to inhibit chondrogenic differentiation of the cells when stimulated with TGFβ-3. Three diketopiperazine (compounds **7**, **8** and **9**) showed the inhibition of hMSC osteogenic differentiation but had no significant activity on chondrogenic differentiation. Compounds **11** and **12** inhibited hMSCs osteogenic differentiation but had no effect on chondrogenesis of the cells.

## 3. Discussion

In this study, we developed a novel high-throughput screening platform for drug discovery in regenerative medicine using automated miniaturized assays based on human mesenchymal stem cells. Unlike previous methodologies [24,35,36], this platform was developed to simultaneously detect osteogenic and chondrogenic differentiation of MSCs in order to decrease the intrinsic variation associated with screening assays involving stem cells and create a body of evidence underpinning hit compounds influencing their behaviour. The different media formulations used to test the compounds allowed for the generation of a wealth of information from a single screening. The assays have the potential to detect inducers of both osteogenic and chondrogenic differentiation when tested in incomplete differentiation media and also identify compounds with the capacity to promote or inhibit differentiation when tested in media formulations containing all differentiation factors.

Considering the limited primary hMSC expansion potential, donor variation and amounts of cells required by high-throughput screenings, a comparison between immortalized hTERT-MSCs and primary hMSCs to differentiate to both osteogenic and chondrogenic lineages was assessed. While hTERT MSCs represent a potentially unlimited population of homogeneous cells, primary cells have limited expansion potential and are subject to donor variation, but still represent a more clinically relevant model. Indeed, primary hMSCs demonstrated a better performance in terms of osteogenic and chondrogenic differentiation. The primary hMSC donor variation was also considered during the assay optimization steps in order to select the most reliable markers to detect bioactivity in the screening platform, finally identified as mineralized calcium for osteogenesis and glycosaminoglycans for chondrogenesis. These cells were therefore chosen as the cell type to be implemented for metabolite screening. Their limited expansion still remains an issue, making the miniaturization a key aspect to allow for the screening of large metabolite libraries. Medium evaporation in the external wells of plates is an issue that can negatively impact readout in high-throughput assays. In this screening, irregular medium evaporation in the 96-well plates used was minimized by using only the 60 inner wells for experimental samples, with outer wells filled with sterile buffer solution. For high-throughput screening investigations involving larger metabolite libraries, the use of gas-permeable plate-sealing membranes is a more appropriate strategy to limit medium evaporation and would enable the use of all available wells for experimental samples.

A comparison of differentiation markers was also performed, in order to develop a reliable and efficient methodology. In cell-based assays, the investigated marker strongly influences the screening length due to the biological time required for the cells to express or synthetize it, while the detection method must be simple enough to allow for automation. For hMSC osteogenic differentiation, the intracellular activity of alkaline phosphatase is the most commonly investigated marker, together with quantitation of the extracellular matrix mineralized calcium [37,38]. Consistent cell donor variation associated with ALP expression and the possibility to detect the inhibition of differentiation through the assessment of calcium mineralization guided the selection of this marker for the platform. Moreover, this assay, coupled with the feeding strategy based on no medium change during the differentiation process, allowed indirect marker detection by calcium depletion in the medium and a streamlined automated process. The detection of hMSC chondrogenic differentiation is based on the quantification of extracellular matrix (ECM) components synthetized by the cells after treatment [39]. Here, a 14-day differentiation process for cells cultured in a high-density monolayer on flat-bottom 96-well plates was used successfully. This longer timeframe enabled the identification of inhibitors and inducers of chondrogenic differentiation. To increase detection sensitivity, two strategies were implemented: cells were fed by medium addition rather than media changes and GAG was quantified in both cell aggregates and medium by adding papain directly to the wells after 14 days incubation. This strategy improved the level of detection, making the high-throughput assay more reliable, with a consistent Z’ factor obtained for several donors.

Marine-derived filamentous fungi are a well-known source of pharmaceutically relevant molecules, previously showing antiviral, antibacterial, anticancer and other bioactivities [40,41]. Particularly promising is the *Penicillium* genus, which showed an extraordinary and unexploited potential to synthetize novel secondary metabolites [42]. The fungal species used in this work, *Penicillium antarcticum*, was isolated from the tunicate *Aplidium pallidum* harvested from the north Atlantic Ocean and is commonly found in the marine environment [43,44] with the potential to synthetize bioactive compounds having been shown previously [45,46]. To investigate new applications for fungal natural products, the pharmacological potential of 12 small molecules isolated from this strain was tested using the developed platform of high-throughput screening. To obtain comparable results in terms of bioactivity, chondrogenic and osteogenic screening were performed using the same cultured cell preparations, split and re-suspended in the appropriate media just prior to plating. Metabolites tested in the presence or absence of primary hMSC differentiation inducers allowed for the evaluation of metabolite potential to stimulate or inhibit hMSC differentiation. Screening results showed that hMSC ossification was inhibited by ethyl esters of itaconic acid **4** and **5** that were able to decrease hMSC marker expression for both osteogenic and chondrogenic differentiation. This inhibitory bioactivity was exerted in vitro in a microenvironment using conditions previously optimised for the induction of hMSC differentiation in the presence of widely used differentiation inducers, such as TGF-β3 for chondrogenesis and dexamethasone for osteogenesis. In this context, the bioactive molecules showed the potential to limit hMSC differentiation while fully stimulated, and therefore may represent promising candidates to limit endochondral ossification in vivo. Even though the detected in vitro bioactivity of compounds **4** and **5** was significant, the in vitro efficacy of these compounds may be further optimised using the developed assays and higher concentrations or even using the factors in combination. The effect of multiple administrations at different time points during in vitro differentiation may also synergise to increase bioactivity and provide a rationale to translate testing to a relevant animal model to assess the retention of bioactivity in vivo. Analogues in their methyl form (compounds **1**–**3**) or carboxylic acid form (**6**) showed less or no bioactivity, highlighting the importance of ethyl esterification of itaconic acid to inhibit hMSC-mediated ectopic calcification. Another class of compounds, diketopiperazines, included in the screening, showed partial bioactivity with the inhibition of osteogenic differentiation by the three molecules containing hydroxyproline. The cell number was assessed post screening to normalize differentiation results. Some variability between treatments was recorded but no trends were evident and the variability is likely to be attributable to the fluctuation expected in these complex cellular assays (Tables S1 and S2). Interestingly, the three methyl esters **1**–**3**, and the two ethyl esters **4**–**5** of itaconic acids tested showed anti-inflammatory bioactivity by decreasing the production of TNF-α by LPS-activated macrophages. The potential of ethyl esters of itaconic acid to inhibit inflammation, in addition to the inhibition of endochondral ossification, is strongly beneficial for the development of therapies to treat diseases such as vascular calcification. In atherosclerosis, the plaque site is characterized by a general inflammation that promotes progenitor cell differentiation and endochondral ossification [47,48,49]. In this context, the compounds tested here could synergise to decrease the expression of pro-inflammatory factors and inhibit endochondral ossification for the amelioration of plaque progression. Further testing is required to evaluate the effect of the itaconate derivatives on the macrophage production of additional pro-inflammatory cytokines, as well as their bioactivity after treatment at different concentrations and timepoints. Compound testing on primary macrophages in addition to the THP1 cell line would also provide a more relevant model for translation to drug development.

## 4. Materials and Methods

### 4.1. Primary and hTERT MSC Culture and Differentiation

Primary hMSCs were isolated from bone marrow obtained from healthy donors, Galway University Hospital, Galway, Ireland. All procedures followed were in accordance with ethical standards of the responsible committees for human experimentation (institutional and national) and the Helsinki Declaration of 1975, as revised in 2000, and informed consent was obtained from all marrow donors included in the study. Isolated hMSCs were expanded in growth medium containing α-minimal essential medium (α-MEM, Life technologies, Carlsbad, CA, USA), 10% foetal bovine serum (FBS, Sigma, St. Louis, MO, USA) and 1% penicillin/streptomycin (P/S, Life technologies) supplemented with 5 ng/mL fibroblast growth factor-2 (FGF-2, Peprotech, Rocky Hill, NJ, USA) and cultured at 37°C, 5% CO_2_. Medium was refreshed three times weekly and cells were subcultured or frozen at 90% confluence in T175 flasks (Sarstedt, Newton, NC, USA).

For osteogenic differentiation, hTERT and primary hMSCs were used up to Passage 4 (P4 –16 and 20 population doublings). Cells were suspended in basic medium (BM) containing phenol red free Dulbecco’s modified eagle medium low glucose (DMEM-LG, Life Technologies), 10% FBS and 1% P/S, and seeded in the 60 inner wells of flat-bottom 96-well plates (Sarstedt). The 24 outer wells were filled with 200 µL of sterile d-PBS to limit evaporation in the experimental wells. Cells were incubated overnight for recovery post-thaw and attachment to the culture surface. To induce differentiation, cells were exposed to an equal volume of OM containing BM supplemented with 0.2 µM dexamethasone (Dex, Sigma), 200 µM ascorbic acid 2-phosphate (AA2p, Sigma) and 20 mM β-glycerophosphate (β-gly, Sigma); this treatment combination was labelled DAG. Untreated control cells were cultured in an equal volume of BM (200 µL). Plates were incubated at 37 °C, 5% CO_2_ with no medium change for 7 or 10 days and analysed for alkaline phosphatase activity and mineralized calcium.

Chondrogenic differentiation was performed using cells up to P4 with hMSCs suspended in incomplete chondrogenic medium (ICM) containing high-glucose phenol red-free DMEM (DMEM-HG, Life Technologies) 1% insulin, transferrin and selenium (ITS^+^) supplement (6.25 μg/mL bovine insulin, 6.25 μg/mL transferrin, 6.25 ng/mL selenous acid, 5.33 μg/mL linoleic acid and 1.25 mg/mL BSA), 50 µg/mL ascorbic acid 2-phosphate, 40 µg/mL l-proline, 100 nM dexamethasone, 1 mM sodium pyruvate and 1% P/S. Fifty thousand cells in 100 µL were seeded in the 60 inner wells of V or flat bottom 96-well plates (Sarstedt) and incubated overnight to enable attachment to flat-bottom plates, or centrifuged at 100 g for 5 min to form cell aggregates in V-bottom plates. The outer 24 wells were filled with d-PBS as described above. To induce differentiation, cells were exposed to an equal volume of complete chondrogenic medium (CCM; ICM with 20ng/mL TGFβ-3). Untreated control cells were treated with ICM and all wells were filled to a final volume of 200 µL. Plates were incubated at 37 °C, 5% CO_2_, 2% O_2,_ with feeding either performed by the exchange of half the medium volume or the addition of 40 µL medium, where stated.

### 4.2. Osteogenic Marker Detection for High Throughput Assay

Primary and hTERT MSC osteogenic differentiation was evaluated by measuring intracellular alkaline phosphatase (ALP) activity and matrix calcium mineralization. To detect ALP activity, the growth medium was discarded, and the cell layer washed twice with 150 µL d-phosphate-buffered saline (PBS; Life technologies). Cells were then lysed by the addition of 50 µL CelLytic M (Sigma) to each well, followed by 15 min incubation at room temperature. Cell lysate (10 µL) was added to 190 µL of ALP detection reagent (*para*-nitrophenylphosphate, *p*-npp, Sigma), and incubated for one hour in the dark at room temperature with absorbance measured at 405nm using a Victor X5 plate reader (PerkinElmer, Waltham, MA, USA). Three technical replicates were performed.

To detect mineralized calcium, two different methods were implemented: in the standard procedure medium was discarded and cells were washed twice with 150 µL D-PBS. HCl (100 µL, 0.5M) was added to solubilize the calcium crystals contained in the cell matrix. After 30 min incubation on a shaker, 10 µL of each sample was assayed in triplicate by adding 190 µL reagent mix from the Calcium Liquicolour Kit (StanBio) and read at 550nm using the Victor plate reader. A method for indirect calcium detection was developed for the HTS format used on the screening platform: no medium change was performed throughout the osteogenic differentiation process and calcium incorporated into the osteogenic matrix was considered equivalent to the calcium depleted from the medium. The amount of mineralized calcium was calculated as the calcium reduction between fresh and spent medium after differentiation. Control wells with medium alone were added to the plates for assessment of the calcium concentration between fresh and spent media. To detect calcium, 10 µL medium from triplicate wells were assayed by adding 190 µL StanBio calcium Liquicolour kit (StanBio) and absorbance was measured at 550 nm using the Victor X5 plate reader. Calcium staining was performed using alizarin red S: differentiated cells were carefully washed with PBS and fixed with ice cold 95% methanol for 10 min. Methanol was then discarded, the cell layer washed once with 150 µL PBS, and 50 µL alizarin red S added for 1 min. The stain was then discarded, cells rinsed twice with 150 µL PBS and incubated with 200 µL PBS at 4 °C overnight to eliminate excess stain before imaging.

### 4.3. Chondrogenic Marker Detection for High Throughput Assay

Chondrogenic differentiation of primary and hTERT MSCs was evaluated as the cells’ ability to synthesize and incorporate sulphated glycosaminoglycans (GAG) in the cartilage matrix. GAG was initially measured in the cellular aggregates: cell medium was discarded, aggregates were washed in PBS and digested using 100 µL papain (*Papaya latex*, Sigma) at 60 °C for 5 h. Ultimately, both secreted and matrix-associated GAG in the cell aggregates was measured in the HTS protocol. Papain was added to wells (100 µg/mL final concentration) containing cell aggregates and differentiation medium and incubated at 60 °C for 5 h. GAG quantification was performed using the dimethylmethylene blue (DMMB) assay for both methods. Briefly, 25 µL of digested samples was added to 20 DMMB and absorbance immediately read at 595 nm using the Victor X5 plate reader.

### 4.4. Automated High Throughput Osteogenic Assay

A streamlined protocol to be run by automated instruments was developed based on the optimization results. Primary hMSCs were subcultured in growth medium until the required cell number was reached, resuspended in BM and seeded (100 µL–12 × 10^3^ per well) in the 60 inner wells of flat-bottom 96-well plates (Sarstedt) using the Janus automated workstation (PerkinElmer). The outer wells were filled with D-PBS as described previously. Plates were incubated overnight for attachment and then treated for differentiation: positive control wells treated with 100 µL OM and negative control with IOM were incubated at 37 °C, 5% CO_2_ for one day. Wells with medium alone (OM and IOM) were included in the plate and used as blanks for the calcium detection assay. After incubation, mineralized calcium was indirectly detected by measuring the amount depleted from the medium during differentiation. Cells and blank media were assayed in triplicate and absorbance was read at 550 nm using the Victor X5 plate reader. Mineralized calcium was calculated as the difference in levels between wells containing fresh and differentiation medium. Cell number was quantified by Hoechst 33342 nuclear staining (Thermo Fisher, Waltham, MA, USA): medium was discarded and the cell layer washed three times with PBS. At the last wash, 25 µL were left in the wells and 25 µL of 10 µg/mL Hoechst 33342 were added. Plates were incubated at room temperature for 10 min and then transferred to the Operetta high-content imaging system (PerkinElmer) for cell number quantification based on nuclei counting.

### 4.5. Automated High Throughput Chondrogenic Assay

Based on the optimization results, a method compatible with automated instruments was established. Primary hMSCs (to P4) were subcultured in growth medium, trypsinized, washed in PBS, resuspended in ICM and seeded at a density of 5 × 10^4^/100 µL medium in the 60 inner wells of flat bottom 96-well plates for assessment using the Janus automated workstation. The outer wells were filled with d-PBS. Plates were incubated overnight to let the cells settle in a high-density monolayer. At day 0, 50 µL medium was discarded and cells were exposed to 40 µL CCM for the induction of chondrogenesis or an equal volume of ICM (negative controls) and incubated at 37 °C, 5% CO_2_, 2% O_2_. To ensure maximal retention of GAG, feeding was performed by medium addition: 40 µL of either ICM or CCM were supplied to the cells at days 2, 4, 7 and 11. After 14 days incubation, 50 µL medium was removed and 10 µL 2.1 mg/mL papain added to each well. Plates were sealed with non-breathable sealing mats (Thermo Scientific) and incubated at 60 °C for 5 h. After digestion, total GAG from the digested aggregates and medium was quantified using the DMMB assay. DNA content was quantified using fluorescent staining: 5 of the sample was added to 50 µL Hoechst 33342 1 µg/mL, tested in duplicate, and fluorescence read immediately at 355 nm excitation, 460 nm emission using the Victor X5 plate reader (PerkinElmer).

### 4.6. Fungal Metabolites Isolation and Characterization

#### 4.6.1. Biological Material

A sample of the tunicate *Aplidium pallidum*, collected the 24th of November 2014 at Corranroo Bay, Galway (53o 8.685′ N; 9o 0.667 W) was used to isolate the associated microbes. The tunicate sample was soaked in isopropyl alcohol for 30 seconds and rinsed three times with sterile water. The inner tissue of the tunicate was then cut using a sterile scalpel to obtain a 2 × 2 mm biomass to inoculate on Potato Dextrose Agar (PDA) plates. The culture medium was prepared using sterile sea water supplemented with streptomycin 50 mg/L and penicillin 50 mg/L to inhibit bacterial growth. Plates were incubated at 28 °C for four weeks and regularly checked for germination. A single colony of a filamentous fungus was transferred to a new agar plate and isolated in pure culture.

Fungal identification was performed using a polyphasic approach based on morphological observation and DNA marker gene amplification and sequencing. The fungal strain morphology was observed on both PDA and SDA media and morphologically identified using a specific taxonomical key [50]. Molecular analyses were performed by sequencing specific genomic regions for *Penicillium* identification: rDNA internal transcribed spacer (ITS), β-tubulin and calmodulin. Taxonomic assignment was based on similarity to reference sequences available at GenBank (nBlast; mismatch 1/-2; gap costs linear). Molecular findings were confirmed morpho-physiologically.

#### 4.6.2. General Procedures

Optical rotation measurements were performed at the Na D line (589.3 nm) with a 5 cm cell at 20 °C on a UniPol L1000 polarimeter (Schmidt + Haensch, Berlin, Germany). UV data were obtained on Chirascan^TM^ V100 (Applied Photophysics, Leatherhead, UK). ^1^H NMR (600 MHz), ^13^C (150 MHz) NMR and 2D spectra were recorded using an Agilent 600 MHz NMR spectrometer equipped with a cryoprobe, in DMSO-d_6_. Chemical shifts are were expressed in δ (ppm) and J values in Hz and referenced to the residual peak of the solvent at δ_H_ 2.50 and δ_C_ 39.5. (+)-HRESIMS data were obtained using an Agilent 1290 Infinity-QTOF 6540 UPLC/MS with electrospray ionization in the positive mode. All DFT calculations have been performed using Gaussian 09W. After optimization of the structure using the B3LYP method at the 6-31 g(g) level, ECD calculations were conducted. Semi-preparative HPLC was performed using a Waters Alliance 2690 chromatography system, equipped with a Waters UV Dual λ Absorbance detector 2487, or by using an Agilent 1260 chromatography system equipped with a G1315B Diode array detector, and G1364 fraction collector. The UV wavelengths used for monitoring separations were 210nm and 254nm. The flow-rate of the mobile phase was 3.0 mL/min for 10 mm columns and 1.0 mL/min for 4.6 mm columns. All solvents used were of HPLC grade. All media components and solvents were purchased either from Fisher Scientific (Dublin, Ireland) and or Sigma Aldrich (Arklow, Ireland). NMR data are reported in Supplementary Table S3, Figures S4–S22, along with qualitative reports for compounds.

#### 4.6.3. Isolation and Structure Elucidation

A 2 L volume of scaled-up fungus broth was freeze-dried which resulted in 10 g of dry biomass material. The biomass was extracted using 1 L of CH_2_Cl_2_/CH_3_OH 1:1, three times over three days. The solvents collected over three days were combined and evaporated under reduced pressure to yield a dry extract of 1.4 g. This extract was suspended in water for partitioning with EtOAc, n-BuOH and CHCl_3_ sequentially. The chloroform soluble layer (0.34 g) was fractionated by MPLC using a reversed-phase C18 Sep-pak (10 g) column (Agilent Bond Elut), with a step gradient elution of 100%, 80%, 60%, 40%, 20% H_2_O/MeOH and 100% MeOH (v/v) at 10 mL/min for 5 min each to produce six subfractions CF1-CF6. The n-BuOH soluble layer (0.21 g) was subjected to Solid Phase Extraction (SPE) using a reversed-phase C18 Sep-pak (10g) column with a step gradient elution of 100%, 50%, 25% H_2_O/MeOH, 100% MeOH and 50% MeOH/CH_2_Cl_2_ (v/v) respectively to produce five subfractions, BV1-BV5. Guided by the reverse phase HPLC-DAD-ELSD based analytical profile, three fractions CF1, BV2, and BV4 were subjected to repeated HPLC purifications. Fraction CF1 (0.2 g) showed the most promising analytical profile in RP-C18 (150 × 4.6 mm, 3.5 μm, Waters XBridge column). This fraction was therefore subjected to semi-preparative reversed-phase HPLC with a gradient elution of 10% MeCN/H_2_O, each containing 0.1% trifluoroacetic acid to 50% MeCN/H_2_O over 35 min (Kromasil-C18 column, 250 × 10 mm, 5 μm, flow 1 mL/min) affording compound **5** (2.3 mg, t_R_ 27.6 min), **2** (1.6 mg, t_R_ 23 min), and **4** (1.7 mg, t_R_ 26.3 min). Other known metabolites **1** (1.6 mg), **3** (0.9 mg), **6** (1.4 mg), **9** (2.2 mg), **7** (1.7 mg), **8** (1.4 mg) and **12** (1.9 mg) were eluted as pure compounds at retention times of 22, 24.6, 23.1, 5.3, 11.8, 14.2 and 20.6 min, respectively. Around 0.8 mg of **1** was also eluted during semi-preparative reversed-phase HPLC of fraction B-V4 using Kromasil-C18 (250 × 10 mm, 5 μm) column gradient of solvent from 5% MeCN/H_2_O each containing 0.1% trifluoroacetic acid for 5 min, and then to 35% MeCN/H_2_O over 35 min at a retention time of 22.9 min along with compound **10** (2.6 mg) at 18.5 min. Under the same conditions, Fraction B-V2 afforded **11** (3.1 mg) at retention time 17.9 min.

Compound **5**: white amorphous powder; ${\left[\alpha \right]}_{D}^{22}$ −6.8° (c 0.23 MeOH); UV (MeOH) λ_max_ (log ε) 208 nm (3.9); ^1^H and ^13^C NMR data in DMSO-d_6_ are listed in the SI; (+)-HRESIMS *m/z* 281.1360 [M + Na]^+^ (calcd. for C_13_H_22_O_5_Na, 281.1359; Δ 0.14ppm).

Compound **2**: white amorphous powder; ${\left[\alpha \right]}_{D}^{22}$ −6.2° (c 0.16 MeOH); UV (MeOH) λ_max_ (log ε) 208 nm (3.8); ^1^H and ^13^C NMR data in DMSO-d_6_ are listed in the SI; (+)-HRESIMS *m/z* 267.1209 [M + Na]^+^ (calcd. for C_12_H_20_O_5_Na, 267.1209; Δ 2.13ppm).

Compound **4**: white amorphous powder; ${\left[\alpha \right]}_{D}^{22}$ −5.0° (c 0.17 MeOH); UV (MeOH) λ_max_ (log ε) 208 nm (3.9); ^1^H and ^13^C NMR data in DMSO-d_6_ are listed in the SI; (+)-HRESIMS *m/z* 281.1361 [M + Na]^+^ (calcd. for C_13_H_22_O_5_Na, 281.1359; Δ 0.53ppm).

### 4.7. Fungal Metabolites Bioactivity Screenings

A number of 12 pure compounds (Figure 4a) isolated from the marine-derived fungus *Penicillium antarcticum* was screened for bioactivity using the developed high-throughput assays. Compounds were tested for cytotoxicity toward immortalized hepatocytes HepG2 on a separate screening before testing for effects on hMSCs, and also tested for their anti-inflammatory potential on LPS-activated THP1 macrophages. The methods and results of the cytotoxicity and anti-inflammatory screenings are described in the Supplementary Materials (Figures S1 and S2).

Using primary hMSCs, the osteogenic bioactivity of the compounds was evaluated using two different scenarios: (i) as osteogenic differentiation inducers, treating the cells in the absence of dexamethasone in the medium (IOM treatment); or (ii) as osteogenic promoters/inhibitors by addition to complete osteogenic medium (OM treatment). Marine compounds dissolved in DMSO were diluted in either IOM or OM to investigate bioactivity and tested at two concentrations (1 and 10 µM) in a final volume of 200 µL medium containing 0.1% DMSO per well. Two columns were assigned for positive and negative controls in each screening plate. Cells (12 × 10^3^) were seeded in flat-bottom 96-well plates and treated with either IOM or OM with marine compounds. Positive control wells for both screening schemes had an equivalent volume of OM + 0.1% DMSO. For the detection of osteogenic inducers IOM + 0.1% DMSO was used as the negative control, whereas BM + 0.1% DMSO was used as the negative control for the detection of osteogenic promoters or inhibitors. Plates were incubated at 37 °C, 5% CO_2_ for 10 days for hMSC differentiation.

The fungal compounds chondrogenic bioactivity was evaluated for the induction of differentiation when tested in ICM-treated cells, with CCM-treated cells as the positive control. The inhibition or promotion of differentiation was evaluated by adding the compounds in CCM (containing TGFβ-3) and comparing to CCM alone as the positive control. The marine compounds dissolved in DMSO were diluted in ICM or CCM and tested at two concentrations (1 and 10 µM) in a final volume of 200 µL per well (0.1% DMSO). Fifty thousand cells in 100 µL were seeded in flat-bottom 96-well plates, incubated overnight and treated with appropriate media containing the marine compound. In each screening plate, two columns were assigned as positive and negative controls, respectively. Positive control wells contained CCM + 0.5% DMSO, while ICM+0.1% DMSO was added to negative control wells. Plates were incubated at 37 °C, 5% CO_2_, 2% O_2_ and cells fed at days 2, 4, 7 and 11 with 40 µL of appropriate media containing the compound to be tested.

### 4.8. Statistical Analyses

The assay quality in high-throughput screenings was assessed through calculation of the Z’ factor [51], a metric that relies on the positive and negative control data to define the assay’s ability to detect bioactivity. The following formula was used to calculate this metric where *σ*_p_ and *σ*_n:_

$$Z\prime =1-3\frac{\sigma p+\sigma n}{\left|µp-µn\right|}$$

are the standard deviations of the positive and negative controls, respectively, and *µ*_p_ and *µ*_n_ are the respective averages. A score of Z’ ≥ 0.5 indicates that the assay was able to discriminate positive hits from background noise on the screening plate. This metric was used during assay development as the threshold to reach at each optimization step, as well as being calculated on all screening plates to confirm reliability. To detect metabolite’s ability to augment differentiation obtained by known compounds or inhibit differentiation, experimental results were compared to the marker level expressed by positive control cells using one-way ANOVA. The detection of metabolite potential to induce differentiation in the absence of appropriate factors was also assessed using one-way ANOVA to detect significance compared to negative control wells. In both cases, data from wells with lower activity compared to the negative control were excluded from the analysis. Statistical analysis for assay optimization steps was performed using two-way ANOVA with Bonferroni post-test analysis with significance defined as *p* < 0.05. All values are reported as the mean and the standard deviation of the mean.

## 5. Conclusions

In summary, a reliable, fully automated screening platform to test metabolite libraries for bioactivity toward human MSCs using cost-effective methodologies was developed to enable drug discovery for regenerative medicine. The assay miniaturization to fit 96-well plates was optimal to test the available small metabolites library using multiple formats: the implemented cell number in the assays was acceptable considering the primary MSCs’ expansion potential and would allow for the screening of bigger libraries. Transition to a 384-well plate format is feasible for the scale-up and testing of larger metabolite libraries. The positive development of such a platform would speed up drug discovery programs in regenerative medicine by furtherly minimizing screening costs and enabling testing at a larger scale. Further investigation on the bioactive metabolites detected in this study (8-hydroxyhexylitaconicacid-1-ethyl, ester and 9-hydroxyhexylitaconicacid-1-ethyl, ester) are currently ongoing in order to understand their involvement in the modulation of stem cell fate and potentially provide a new drug candidate for currently untreated diseases.

## Supplementary Materials

The following are available online at https://www.mdpi.com/1660-3397/18/4/192/s1, Figure S1: Marine compound cytotoxicity assessment, Figure S2: Marine compound anti-inflammatory bioactivity and viability of THP1 cells after treatment. Table S1: Number of cells counted for osteogenic screening, Table S2: DNA measured for chondrogenic screening. NMR and MS Data for compounds **5**, **2** and **4** and ECD spectra for **5**.
