# Supplementary material for: A Novel High-Throughput Screening Platform Identifies Itaconate Derivatives from Marine Penicillium antarcticum as Inhibitors of Mesenchymal Stem Cell Differentiation
Source: Mar Drugs. 2020 Apr 5;18(4):192. doi: 10.3390/md18040192 (PMC7230868; doi:10.3390/md18040192)
Supplement: Supplementary file 1 [file marinedrugs-18-00192-s001.pdf]

Supplementary Materials

A novel high throughput screening platform identifies itaconate derivatives from marine *Penicillium antarcticum* as inhibitors of mesenchymal stem cell differentiation

Pietro Marchese[1], Nipun Mahajan[2], Enda O'Connell[3], Howard Fearnhead[4], Maria Tuohy[5], Janusz Krawczyk[6], Olivier P. Thomas[2], Frank Barry[1] and J Mary Murphy[1]

[1] National University of Ireland Galway; Regenerative Medicine Institute; School of Medicine, Galway, Ireland
[2] National University of Ireland Galway; Marine Biodiscovery and Ryan Institute; School of Chemistry, Galway, Ireland
[3] National University of Ireland Galway; Genomics and Screening Core; National Centre for Biomedical Engineering Science, Galway, Ireland
[4] National University of Ireland Galway; Pharmacology and Therapeutics; School of Medicine, Galway, Ireland
[5] National University of Ireland Galway; Molecular Glycobiotechnology; School of Natural Sciences, Galway, Ireland
[5] National University of Ireland Galway; Galway University Hospital; School of Medicine, Galway, Ireland
* Correspondence: p.marchese1@nuigalway.ie

1. Material and Methods

1.1 High throughput screening cytotoxicity of the metabolite library

To assess the extracts' cytotoxicity, a miniaturized high throughput assay was performed using an automated workstation (Perkin Elmer). A liver cancer cell line (HepG2) was treated with the compounds at 1 and 10μM. Pure compounds were dissolved in dimethyl sulfoxide (DMSO, Sigma-Aldrich) and diluted in cell culture medium to reach the working concentrations needed. The final DMSO concentration was kept at 0.1% for all treatments. Cells were cultured in Basic Medium (BM) containing: αMEM and 1% penicillin/streptomycin (Thermofisher), plus 10% FBS (Sigma Aldrich). Culture flasks were maintained at 37°C, 5% CO_2 in a humidified atmosphere. The screening was performed by seeding $5x10^3$ cells in flat-bottom 96-well plates. After overnight incubation, treatment was performed by adding 100μL of BM with either diluted compounds or controls; all conditions were tested in triplicate. Cells were then incubated at 37°C, 5% CO_2 for 72 hours. Positive control cells were treated with 0.1% DMSO and negative controls with 10% DMSO to induce cytotoxicity. Cell viability after treatment was evaluated by measuring reduction of the vital reagent resazurin (AlamarBlue - Thermofisher). A 10% v/v of reagent was added to the wells and plates incubated for 6 hours at 37°C, 5% CO_2 to let the cells metabolize the reagent. After incubation, fluorescence was measured at 531nm excitation wavelength, 572nm emission wavelength.

1.2 High throughput screening for anti-inflammatory activity of the metabolite library

A miniaturized assay to investigate the compounds' anti-inflammatory bioactivity was developed in 96-well plates using a macrophage cell line (THP1). Cells were cultured in RPMI growth medium with 10% FBS and 1% penicillin/streptomycin (P/S, Life technologies), and incubated at 37°C, 5% CO_2. Medium was refreshed three times weekly and cells were sub-cultured or frozen at 90% confluence in T175 flasks (Sarstedt). To perform the assay, cells were resuspended ($5x10^5$/ml) in growth medium containing 1μg/ml LPS to trigger the inflammation and seeded (100μl) in U-bottom 96-well plates. Negative control cells were resuspended in growth medium without LPS and seeded in three wells of

each plate. Compounds to test for bioactivity were diluted in growth medium and cells were treated with 100µl medium containing 1µM of each compound. Positive and negative control cells were treated with growth medium only and DMSO concentration was kept at 0.1% in both treatment and control wells. Plates were incubated for 6 hours at 37°C, 5% CO_2 and then processed to measure the cellular inflammation state and cell viability after treatment. Plates were centrifuged for 5 min at 150g and medium was collected into a fresh 96-well plate without disturbing the cell pellet. Cells were treated with 200ul growth medium containing 10% alamarBlue (Thermofisher), the medium was gently pipetted 5 times to resuspend the cells and plates were incubated 6 hours at 37°C, 5% CO_2. To assess viability, cell reduction of the resazurin-based viable stain was detected using the Viktor plater reader measuring fluorescence at 531nm excitation and 572nm emission. Inflammation was measured as the levels of the pro-inflammatory cytokine TNF-α in the media using the human TNF-α DuoSet ELISA (R&D systems), following the manufacturer instructions. The Viktor plate reader (PerkinElmer) was used for absorbance measurement at 450nm and 495nm wavelengths.

2 Results

2.1 Metabolite library cytotoxicity assessment

Cytotoxicity of the marine metabolites was measured before screening for hMSC bioactivity in order to exclude cytotoxic compounds or concentrations from the screening (Figure S1). All compounds tested at 1 and 10µM did not show any significant cytotoxic activity on HepG2 cells compared to untreated control cells and therefore both concentrations of all compounds were included for screening for hMSC bioactivity detection.

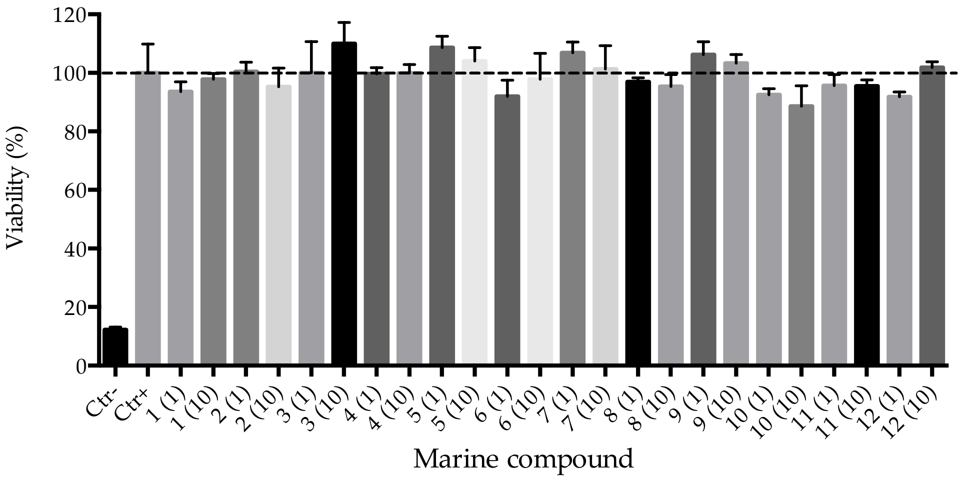

Figure S1: Marine compound cytotoxicity assessment. Five thousand hepatocytes (HepG2) were seeded in 96-well plates and treated with the marine metabolite library. Compounds (1-12) were suspended in DMSO and diluted in cell growth medium to generate concentrations of 1µM (1) and 10µM (10). After treatment, cells were incubated for 72 hours at 37°C, 5% CO_2 Positive control cells were treated with an equal volume of growth medium containing 0.1% DMSO while negative control cells were treated with 10% DMSO to kill the cells. After incubation, 10% v/v resazurin based dye (alamarBlue) was added to the cell medium and incubated for 6 hours. Fluorescence was measured at 531nm excitation, 572nm emission. Cytotoxicity was compared to the positive control treated cells. Results are presented as the mean ± SD of 3 technical replicates, * = $p \leq 0.05$ calculated using ANOVA one-way with Bonferroni post-test

2.2 Cell number or DNA levels for hMSCs post treatment with fungal metabolites

Table S1. Average cell counts post osteogenic screening

	Osteogenic medium				Incomplete Osteogenic medium			
	**1μM**		**10μM**		**1μM**		**10μM**	
**Compound**	Mean	SD	Mean	SD	Mean	SD	Mean	SD
Ctr-	9593	759	9593	1065	9310	7	8866	525
Ctr+	10529	2991	10524	157	15071	440	11943	1104
1	12291	567	9126	754	13098	649	10191	249
2	13979	283	11505	1872	15361	164	12191	753
3	10796	503	11536	1574	9342	425	12270	254
4	11327	991	12259	1507	14144	592	12020	626
5	11368	67	11790	1499	12117	1141	14179	164
6	13546	656	11447	612	14116	1343	14155	1929
7	11281	609	10030	259	12718	1022	10174	1022
8	12027	261	12180	112	14344	589	13374	515
9	9442	1716	10212	1089	14485	625	14401	761
10	15788	37	12741	749	13050	231	10745	261
11	13356	1343	11721	1393	12945	418	11737	1291
12	11563	479	12147	496	14074	477	11953	1194

Cell number is represented as the mean of three wells ± standard deviation (SD)

Table S2. DNA levels measured post chondrogenic screening

	Complete Chondrogenic Medium				Incomplete Chondrogenic Medium			
	**1μM**		**10μM**		**1μM**		**10μM**	
**Compound**	Mean	SD	Mean	SD	Mean	SD	Mean	SD
Ctr-	538	17	568	15	593	41	617	26
Ctr+	604	7	594	3	556	86	588	26
1	575	25	563	19	577	334	595	15
2	628	12	601	15	610	18	671	14
3	574	11	537	49	589	329	579	44
4	624	27	629	18	629	32	641	46
5	604	18	572	18	606	30	651	16
6	620	14	607	22	584	20	638	19
7	639	9	599	66	541	48	608	23
8	578	9	569	15	533	39	619	9
9	587	7	584	10	558	42	640	10
10	578	13	570	17	601	16	631	7
11	586	16	528	55	566	79	604	13
12	545	56	529	45	544	32	593	16

DNA is represented in μg/well as the average of three wells ± standard deviation (SD)

2.3 Metabolite library anti-inflammatory assessment

The marine compound library was tested on a separate screening to detect potential anti-inflammatory properties. Anti-inflammatory properties against LPS activated THP1 macrophages were recorded for all the itaconic acid derivates tested at 1μM (Figure S2).

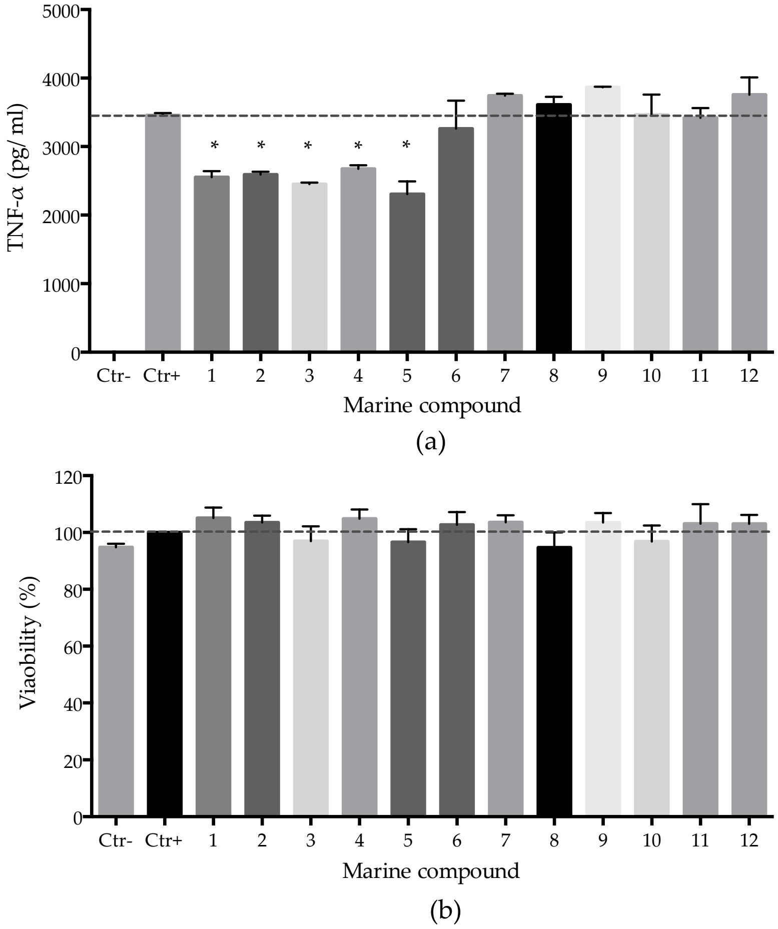

Figure S2: Marine compound anti-inflammatory bioactivity and viability of THP1 cells after treatment. Macrophages THP1 were exposed to 1μg/ml LPS to trigger inflammation and seeded ($5x10^4$) in U-bottom 96-well plates. Compounds of the marine metabolite library, dissolved in DMSO, were diluted in cell growth medium and added to the cells to a final concentration of 1μM. Negative control cells (Ctr-) were seeded without LPS or exposed to the marine compound treatment while positive control cells (Ctr+) were only exposed to LPS. Plates were incubated for 6 hours at 37°C, 5% CO_2 and processed for anti-inflammatory testing and cell viability. (a) The inflammation state was measured as the medium level of TNF-α: plates were centrifuged, medium collected and tested using the hTNF-α DuoSet ELISA. Absorbance at 450nm and 595nm wavelengths was measured. (b) To evaluate viability after treatment, cells were resuspended in fresh growth medium added to 10% alamarBlue and incubated for 6 hours 37°C, 5% CO_2. Fluorescence was then measured at 531nm excitation, 572nm emission. Cell viability after treatment was compared to positive and negative control cells. Results are presented as the mean ± SD of 3 technical replicates, * indicates $p \leq 0.05$ calculated using ANOVA one-way with Bonferroni post-test.

2.4 Isolation and structure elucidation of selected fungal metabolites

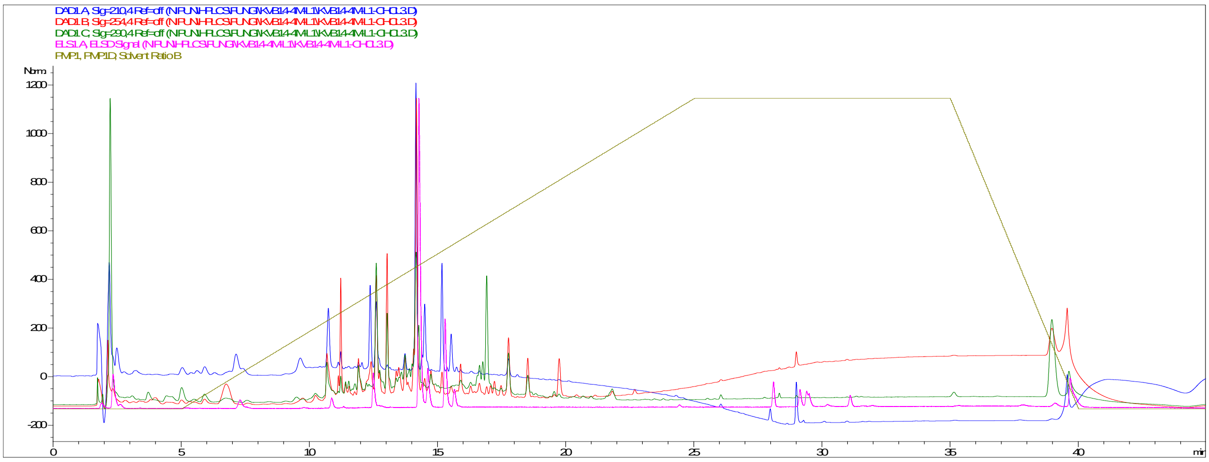

Figure S3: HPLC-DAD-ELSD C18 standard analytical profiles of the $CHCl_3$ fraction

Table S3. ^{1}H (600 MHz) and ^{13}C (150 MHz) NMR Data for compounds **5**, **2** and **4** in DMSO-d_6

	**5**		**2**		**4**	
Atom no.	δ_C, mult.	δ_H mult. (J in Hz)	δ_C, mult.	δ_H mult. (J in Hz)	δ_C, mult.	δ_H mult. (J in Hz)
1	172.5, C_q		173.1, C_q		172.6, C_q	
2	46.3, CH	3.37 [a]	46.2, CH	3.39 [a]	46.2, CH	3.39 [a]
3	139.0, C_q		138.9, C_q		139.1, C_q	
4	167.1, C_q		167.1, C_q		167.1, C_q	
5	30.5, CH_2	1.58,m<br>1.73,m	30.4, CH_2	1.56,m<br>1.74,m	30.4, CH_2	1.58, m<br>1.75, m
6	23.2, CH_2	1.20, m<br>1.33, m	23.2, CH_2	1.18, m<br>1.31, m	27.1, CH_2	1.20, m[a]
7	36.2, CH_2	1.26, m<br>1.33, m	36.2, CH_2	1.23, m<br>1.31, m	38.8, CH_2	1.28, m[a]
8	70.7, CH	3.28, br s	70.7, CH	3.24, br s	25.0, CH_2	1.24, m[a]<br>1.32, m[a]
9	29.8, CH_2	1.27, m<br>1.34, m	29.8, CH_2	1.24, m<br>1.31, m	65.6, CH	3.53, m
10	10.0, CH_3	0.83, t (7.0)	10.0, CH_3	0.82, t (7.4)	23.6, CH_3	1.01 d (6.1)
11	126.0, CH_2	6.20, d (2.0)<br>5.71, d (2.0)	126.3, CH_2	6.21,s<br>5.72,s	126.0, CH_2	6.20,s<br>5.70,s
12	60.0, CH_2	4.05, q (7.0)	51.5, CH_3	3.57,s	60.0, CH_2	4.03, q (7.1)
13	14.0, CH_3	1.14, t (7.0)	-		14.0, CH_3	1.13, t (7.1)

[a] Signals overlapped.

Qualitative Compound Report

**Data File**	C-F1-P21.d	**Sample Name**	C-F1-P21
**Sample Type**	Sample	**Position**	P1-A3
**Instrument Name**	Instrument 1	**User Name**	
**Acq Method**	Biodiscovery Pos standard.m	**Acquired Time**	5/16/2016 5:43:31 PM
**IRM Calibration Status**	Success	**DA Method**	C-F1-P21 method.m
**Comment**			

Sample Group **Info.**
Acquisition SW 6200 series TOF/6500 series
Version Q-TOF B.05.01 (B5125)

Compound Table

Compound Label	RT	Mass	Abund	Formula	Tgt Mass	Diff (ppm)
Cpd 1: C13 H22 O5	6.39	258.1468	3698	C13 H22 O5	258.1467	0.14

MS Zoomed Spectrum

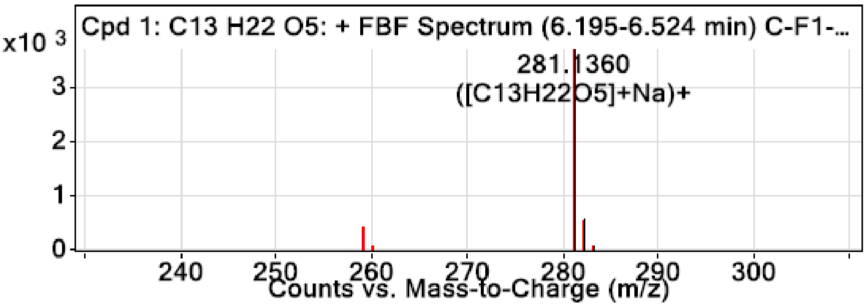

--- End Of Report ---

Figure S4. UHPLC-qToF analysis of Compound **5** in (+)-ESI mode

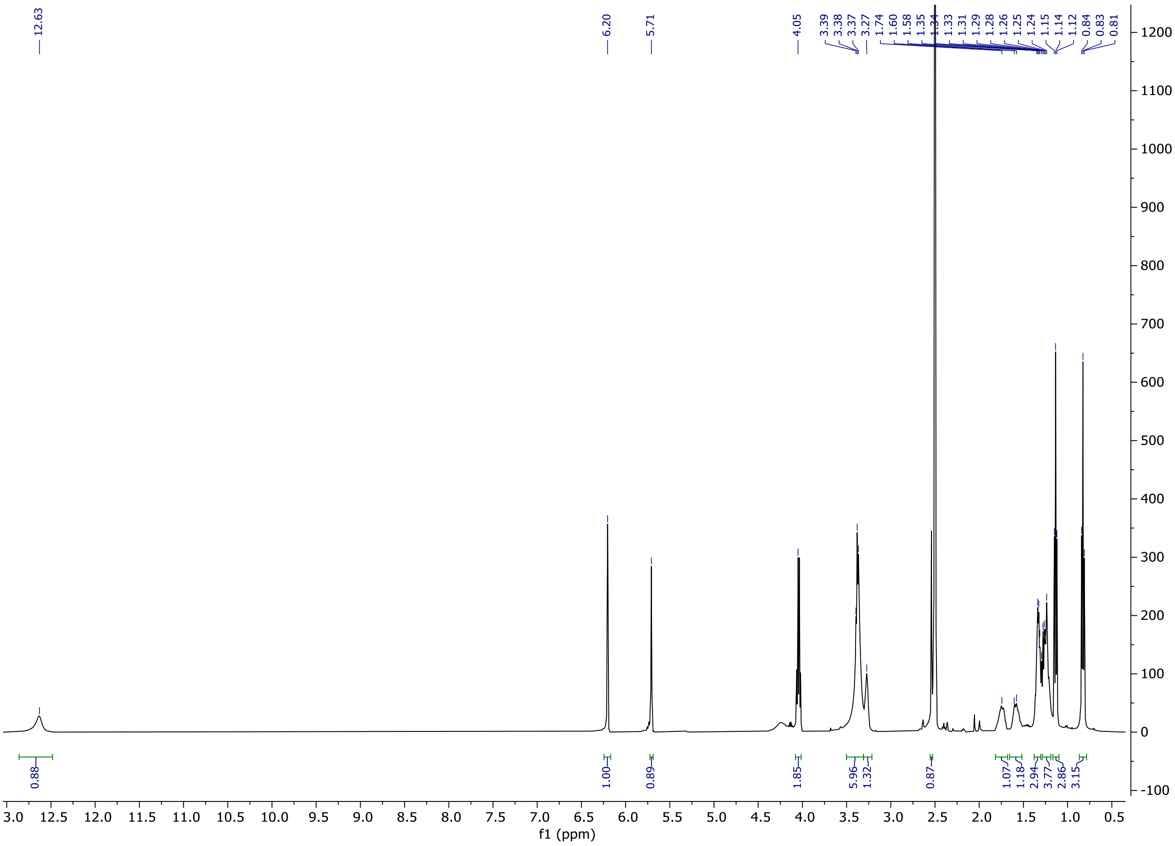

Figure S5. ^{1}H NMR spectrum of Compound **5** in DMSO-d_6 (600 MHz)

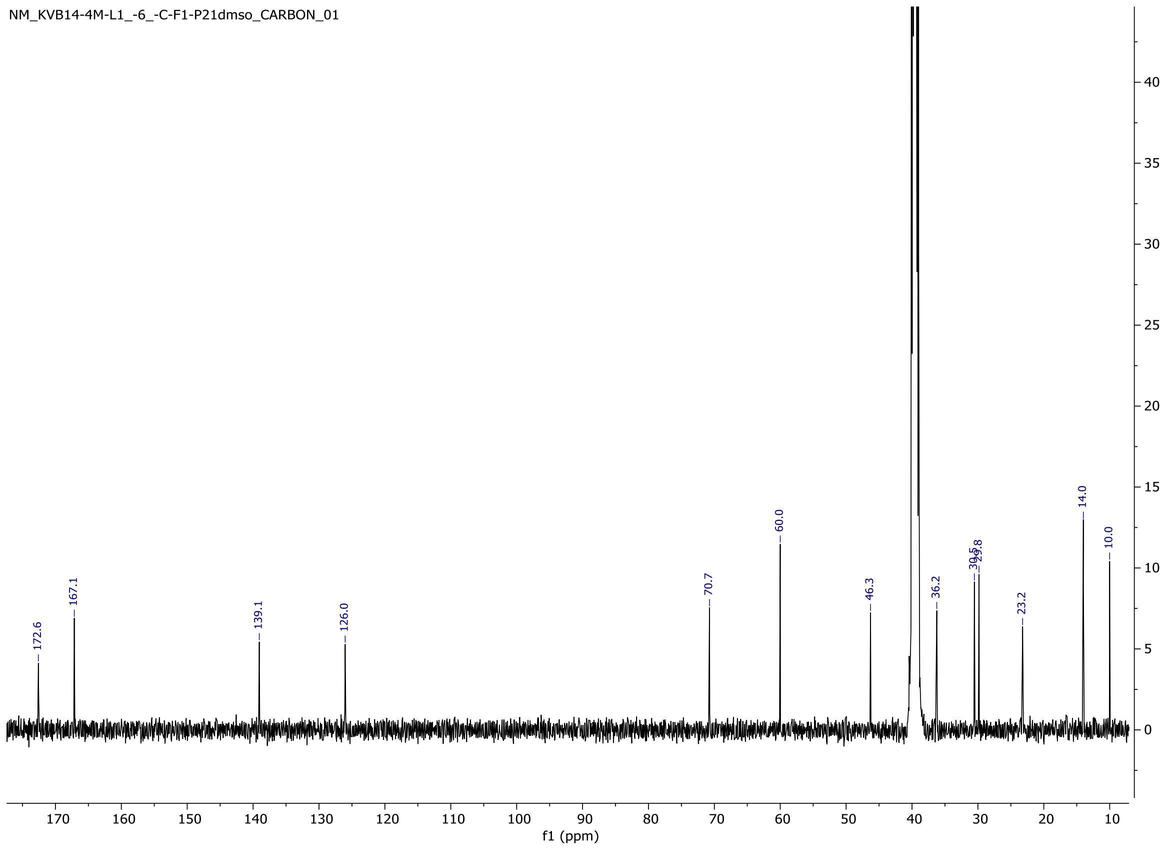

Figure S6. ^{13}C NMR spectrum of Compound **5** in DMSO-d_6 (150 MHz)

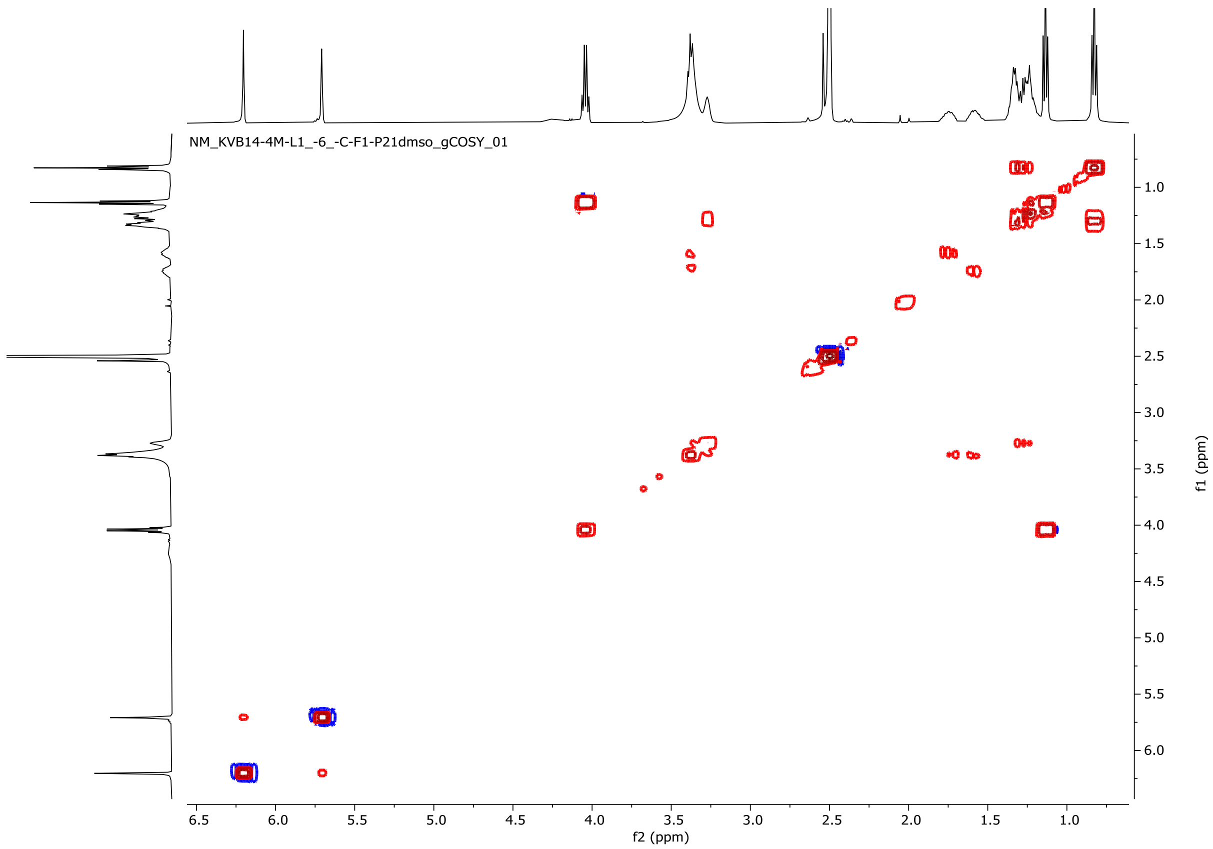

Figure S7. COSY NMR spectrum of Compound **5** in DMSO-d_6 (600 MHz)

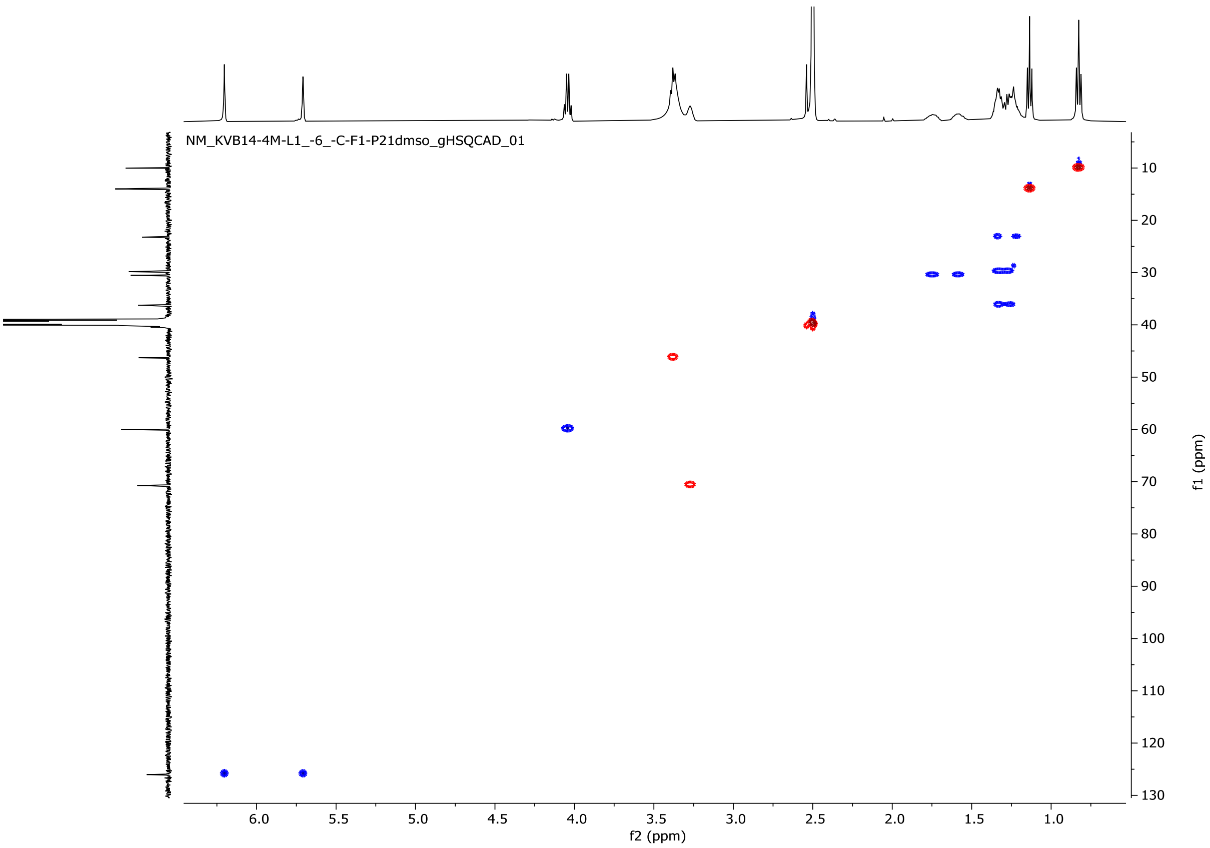

Figure S8. HSQC NMR spectrum of Compound **5** in DMSO-d_6 (600 MHz)

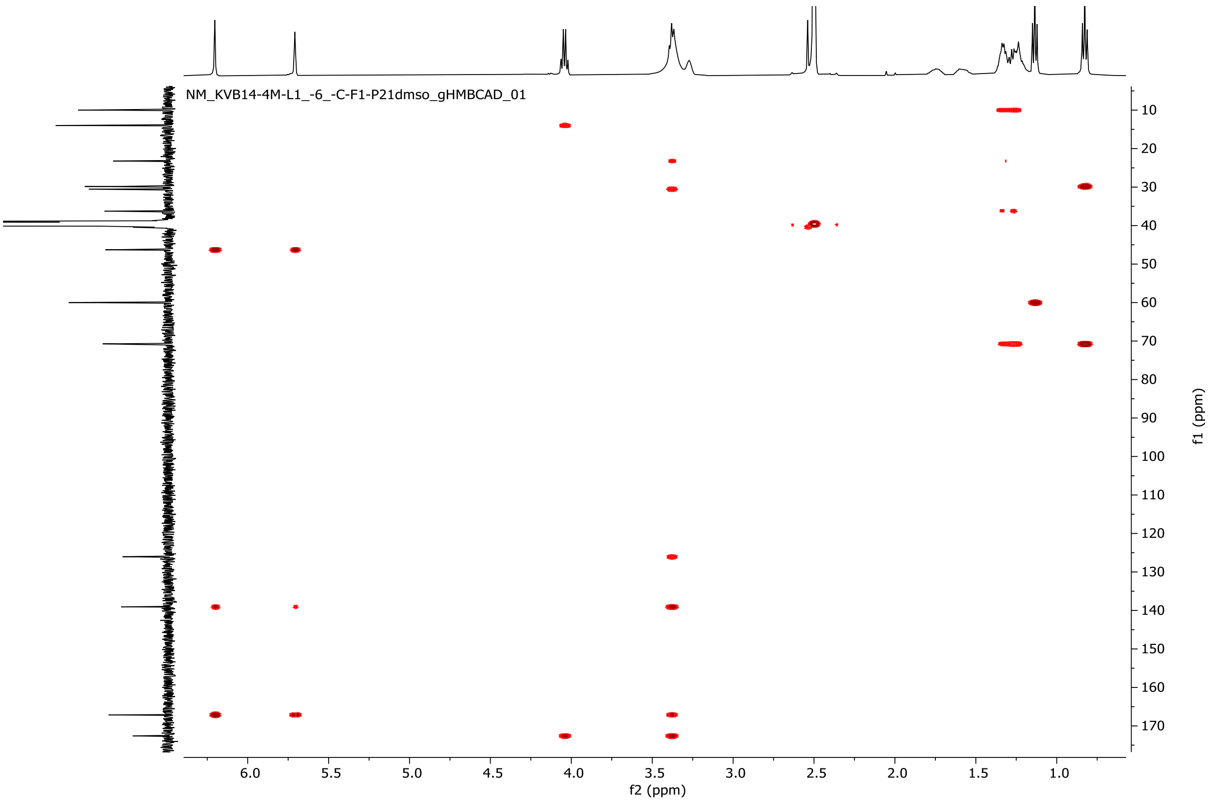

Figure S9. HMBC NMR spectrum of Compound **5** in DMSO-d_6 (600 MHz)

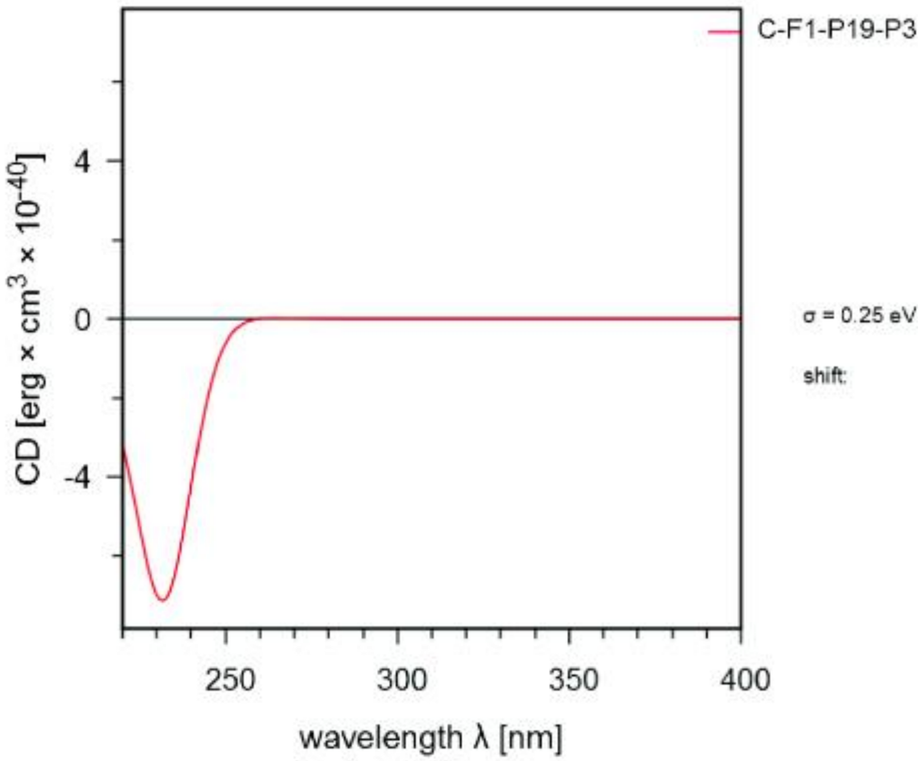

Predicted ECD spectrum

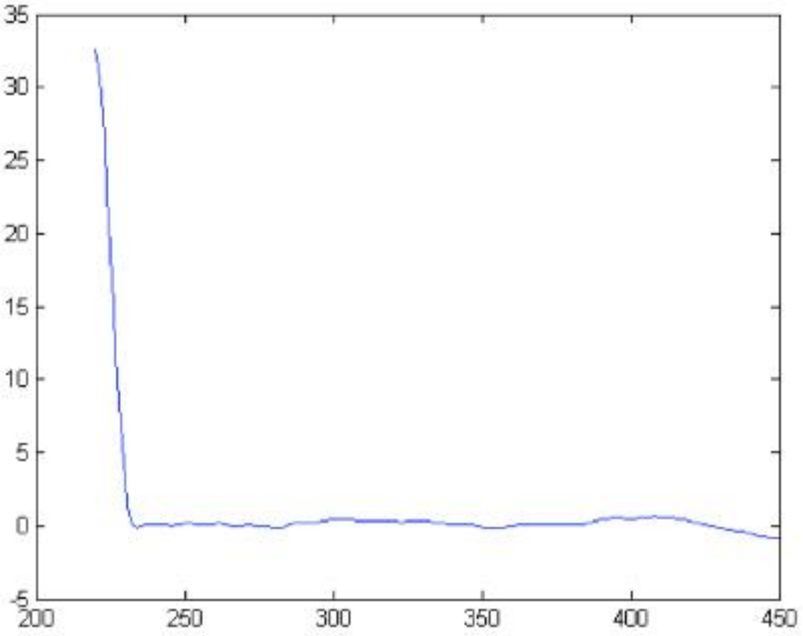

Experimental ECD Spectra

Figure S10. Comparison between the calculated spectrum of both epimers at C-8 having a 2*S* configuration and the experimental spectrum of compound **5**.

Qualitative Compound Report

**Data File**	CF1-P19-P3msms.d	**Sample Name**	CF1-P19-P3msms
**Sample Type**	Sample	**Position**	P1-A2
**Instrument Name**	Instrument 1	**User Name**	
**Acq Method**	Biodiscovery auto-msms CE 20.m	**Acquired Time**	5/26/2016 5:18:14 PM
**IRM Calibration Status**	Success	**DA Method**	C-F1-P21 method.m
**Comment**			

**Sample Group**		**Info.**
**Acquisition SW Version**	6200 series TOF/6500 series Q-TOF B.05.01 (B5125)	

Compound Table

Compound Label	RT	Mass	Abund	Formula	Tgt Mass	Diff (ppm)
Cpd 1: C12 H20 O5	4.388	244.1316	96736	C12 H20 O5	244.1311	2.13

Compound Label	*m/z*	RT	Algorithm	Mass
Cpd 1: C12 H20 O5	267.1209	4.388	Find By Formula	244.1316

MS Zoomed Spectrum

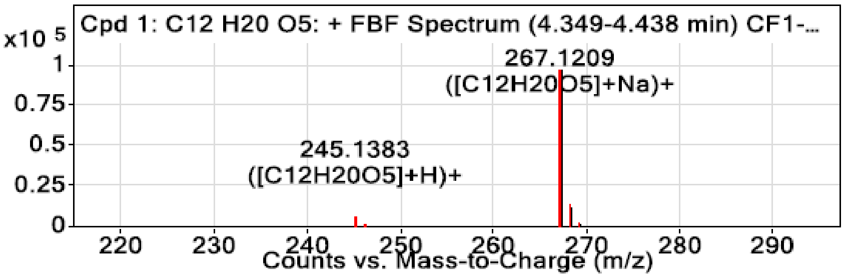

--- End Of Report ---

Figure S11. UHPLC-qToF analysis of Compound **2** in (+)-ESI mode

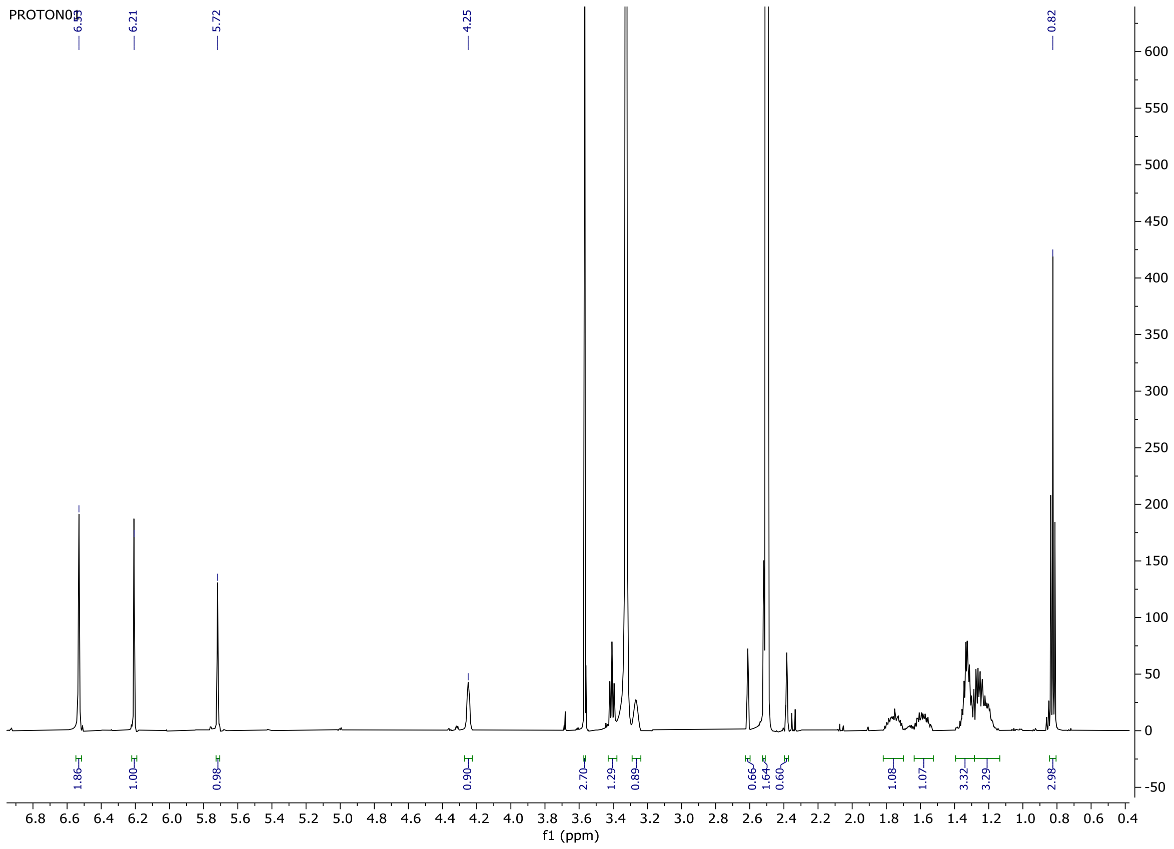

Figure S12. ^{1}H NMR spectrum of Compound **2** in DMSO-d_6 (600 MHz)

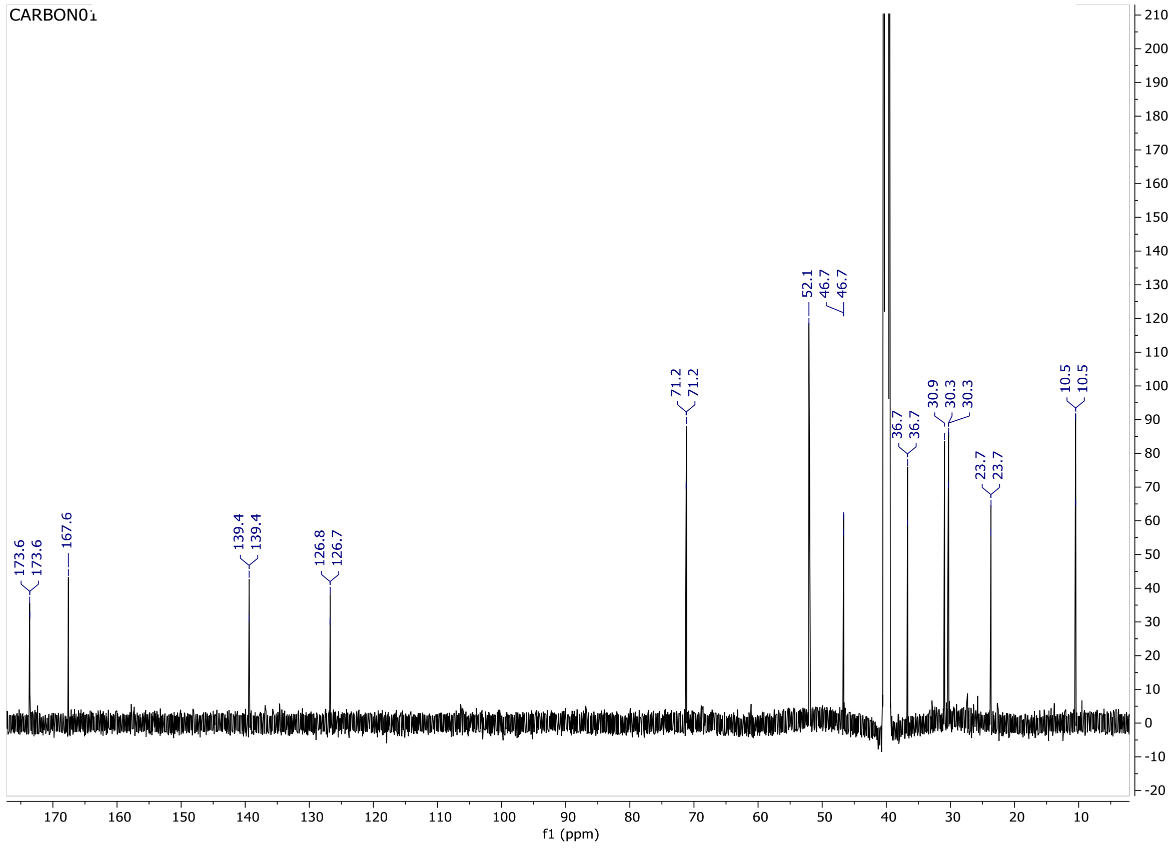

Figure S13. ^{13}C NMR spectrum of Compound **2** in DMSO-d_6 (150 MHz)

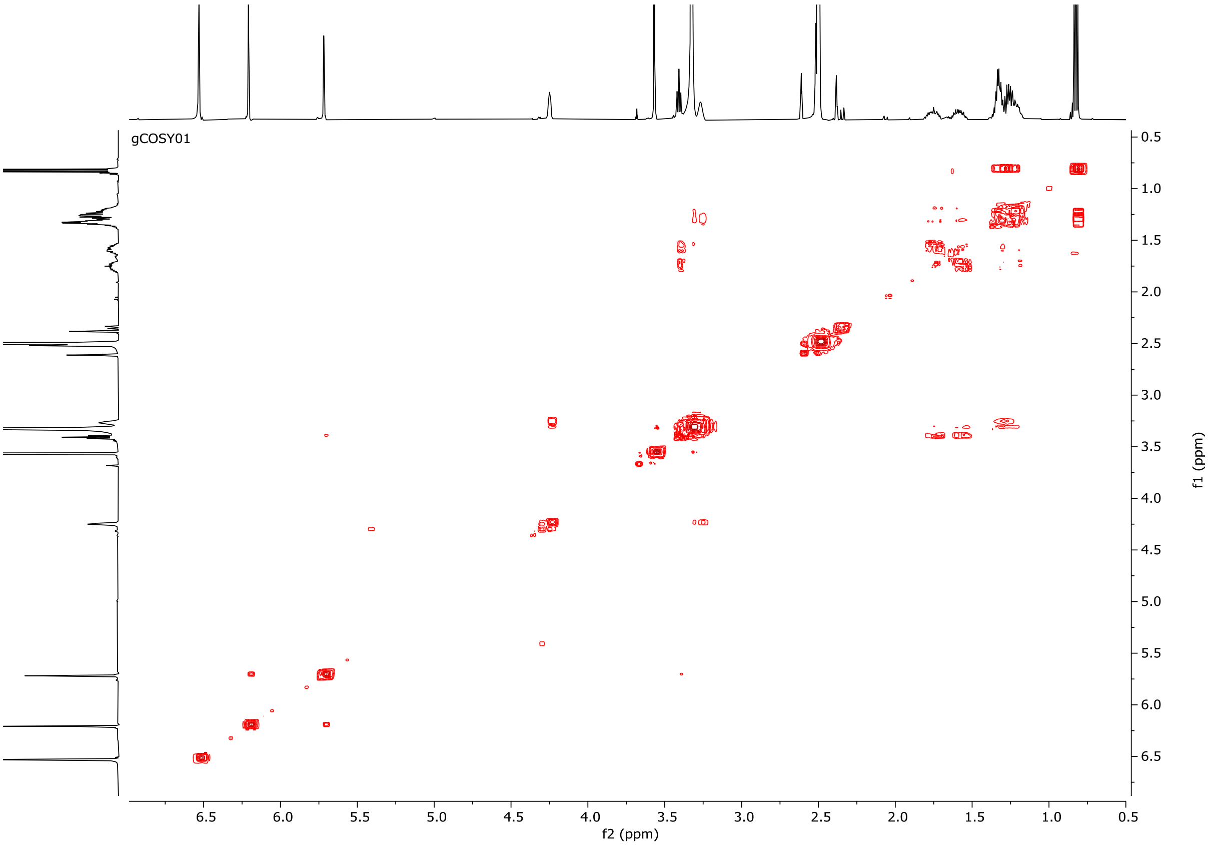

Figure S14. COSY NMR spectrum of Compound **2** in DMSO-*d*$_6$ (600 MHz)

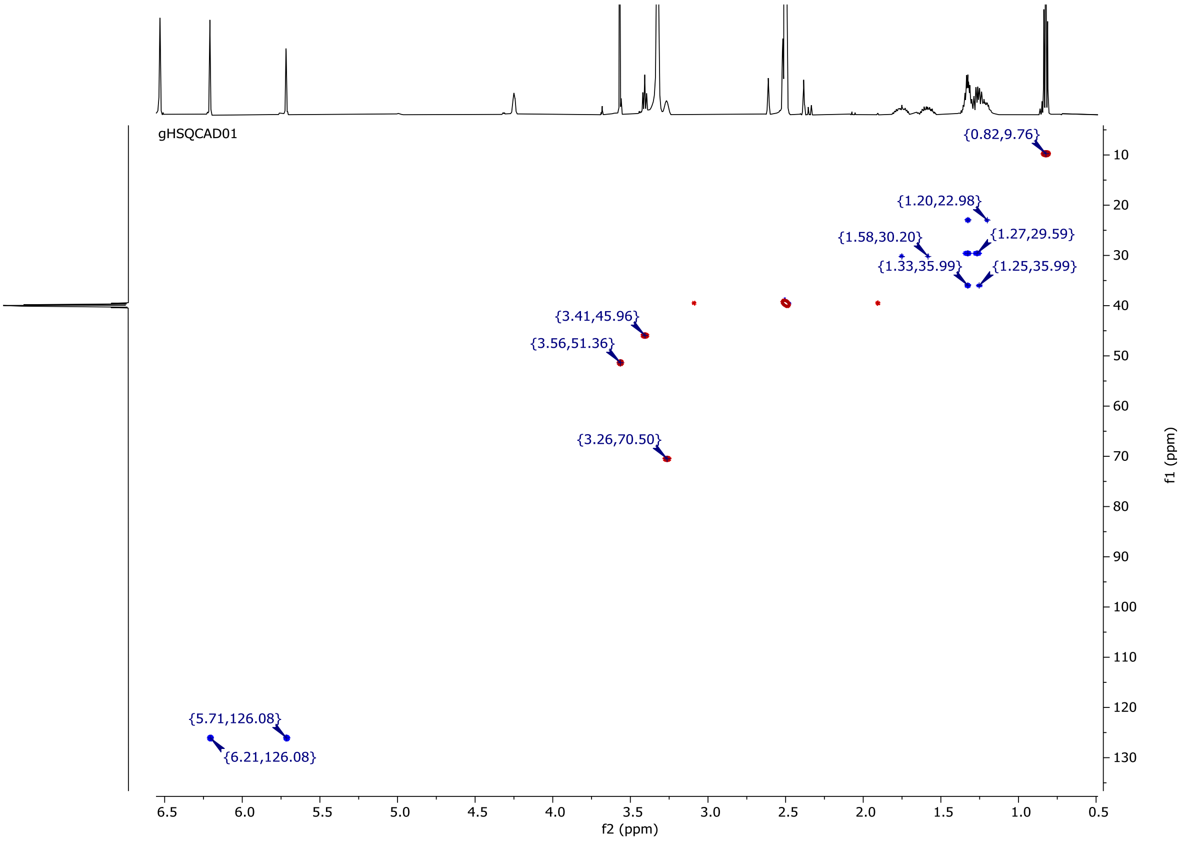

Figure S15. HSQC NMR spectrum of Compound **2** in DMSO-*d*$_6$ (600 MHz)

134

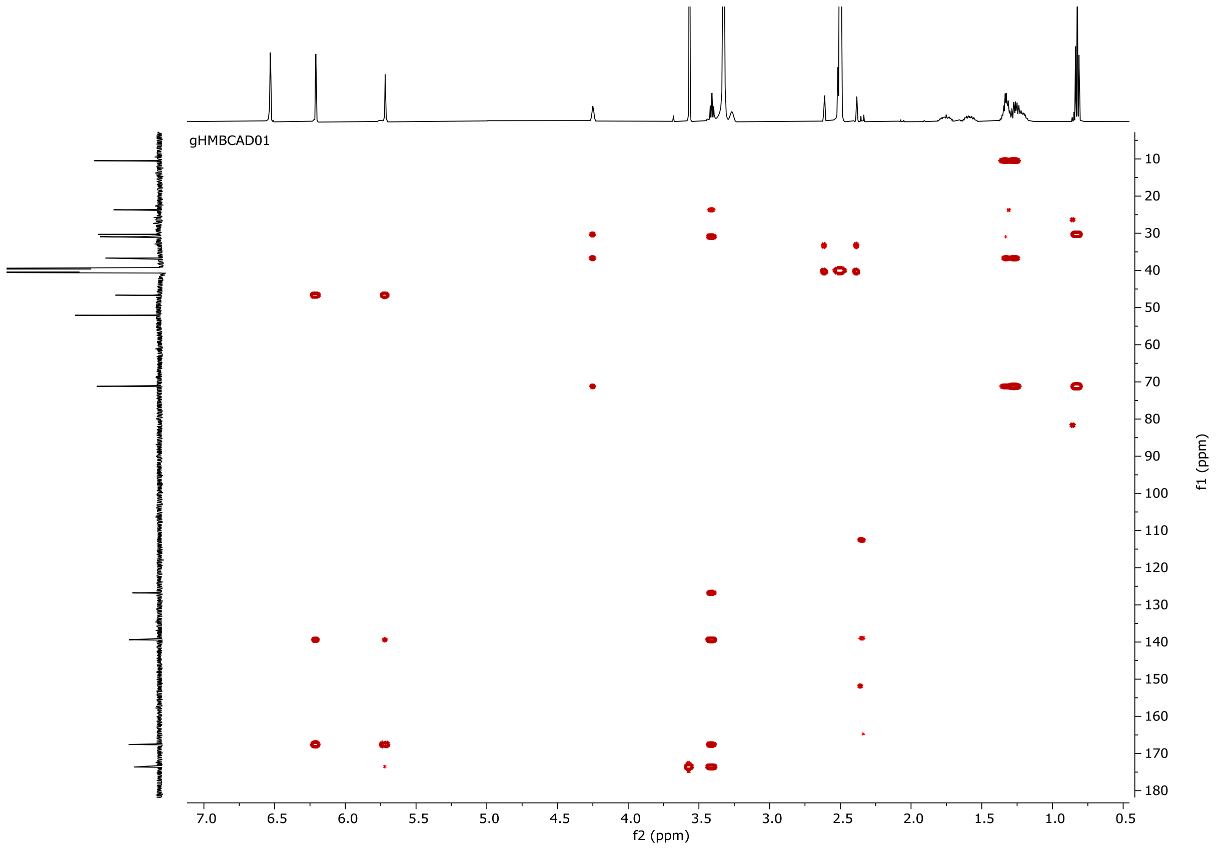

Figure S16. HMBC NMR spectrum of Compound **2** in DMSO-d_6 (600 MHz)

Qualitative Compound Report

**Data File**	C-F1-P20.d	**Sample Name**	C-F1-P20
**Sample Type**	Sample	**Position**	P1-A2
**Instrument Name**	Instrument 1	**User Name**	
**Acq Method**	Biodiscovery Pos standard.m	**Acquired Time**	5/16/2016 5:28:36 PM
**IRM Calibration Status**	Success	**DA Method**	C-F1-P21 method.m
**Comment**			

Sample Group **Info.**
Acquisition SW 6200 series TOF/6500 series
Version Q-TOF B.05.01 (B5125)

Compound Table

Compound Label	RT	Mass	Abund	Formula	Tgt Mass	Diff (ppm)
Cpd 1: C13 H22 O5	6.377	258.1469	3071	C13 H22 O5	258.1467	0.53

MS Zoomed Spectrum

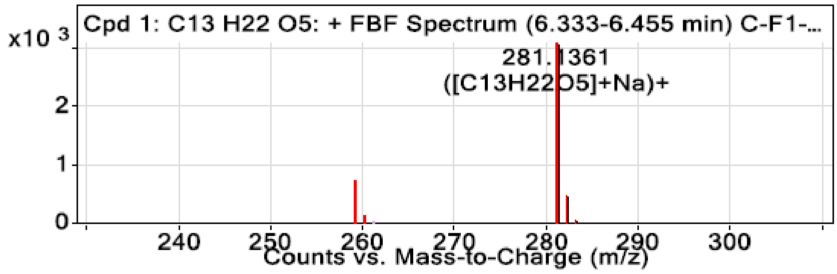

--- End Of Report ---

Figure S17. UHPLC-qToF analysis of Compound **4** in (+)-ESI mode

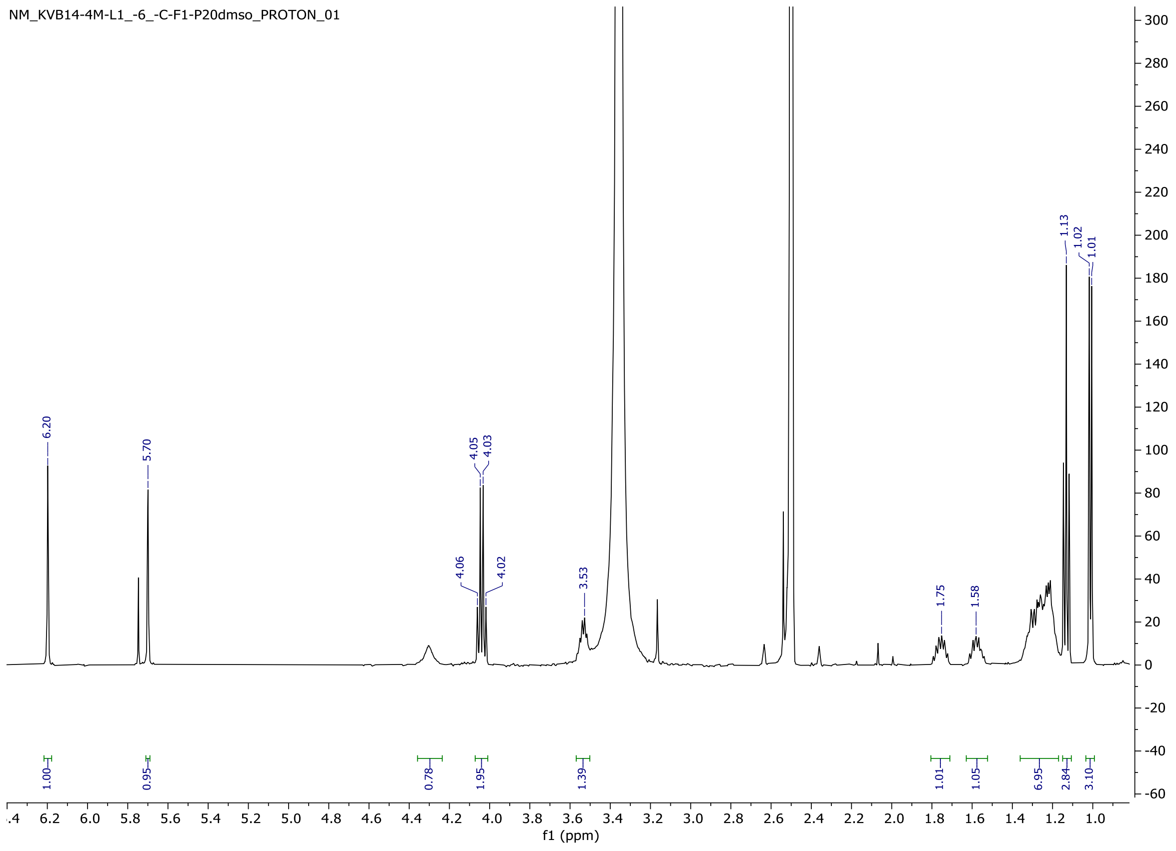

Figure S18. ^{1}H NMR spectrum of Compound **4** in DMSO-d_6 (600 MHz)

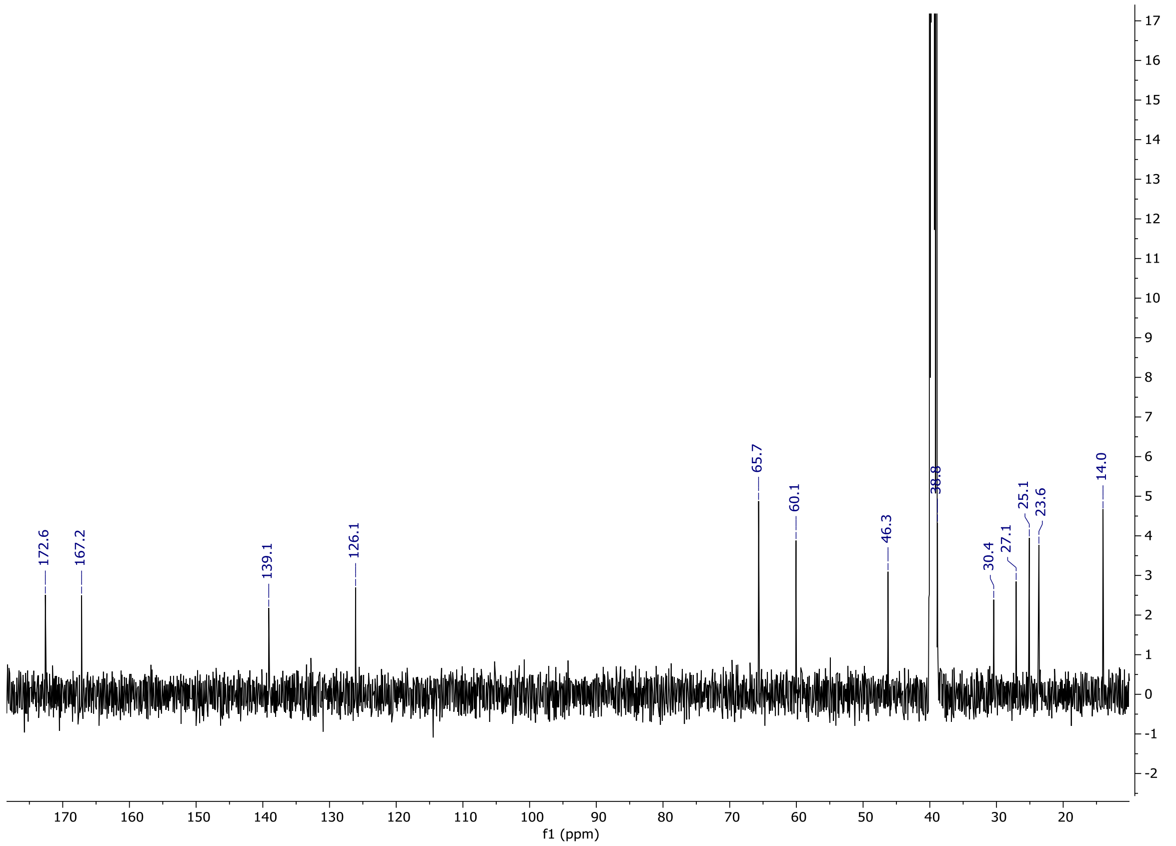

Figure S19. ^{13}C NMR spectrum of Compound **4** in DMSO-d_6 (150 MHz)

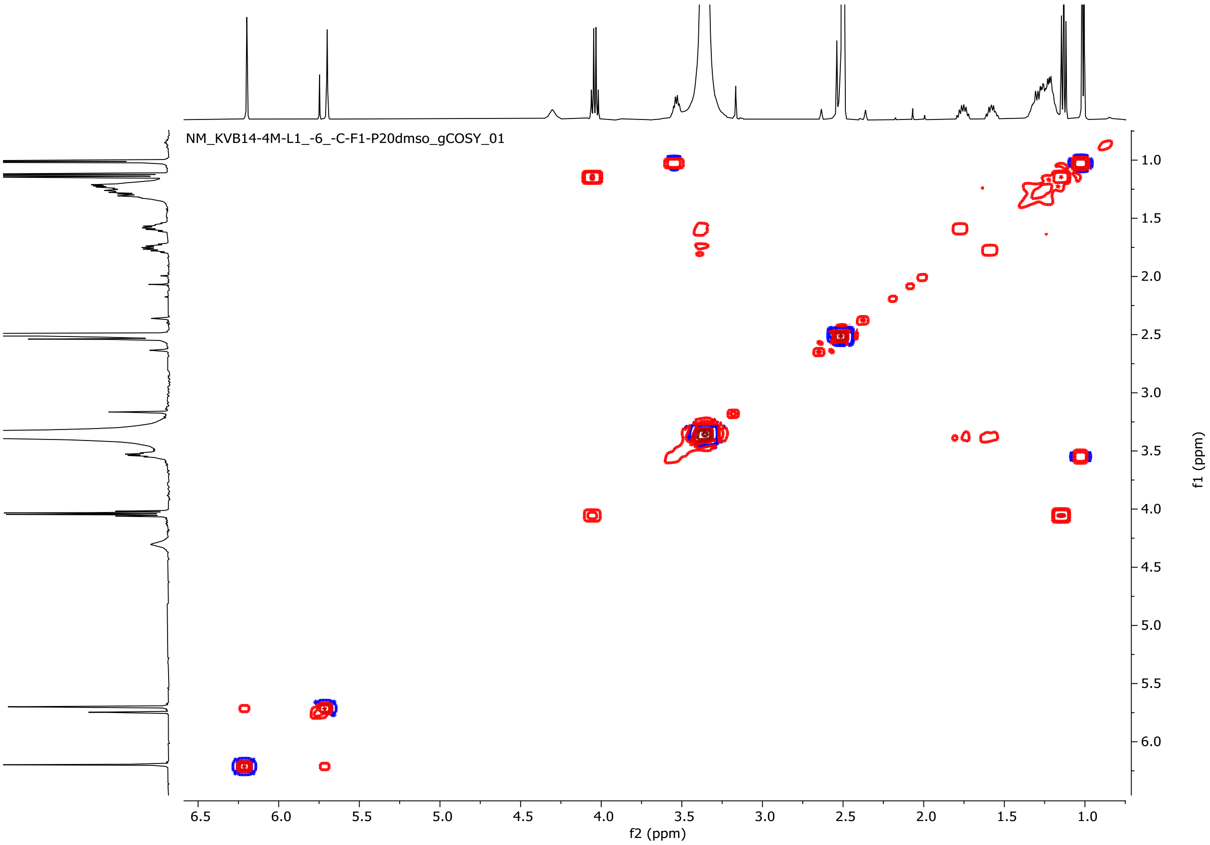

Figure S20. COSY NMR spectrum of Compound **4** in DMSO-d_6 (600 MHz)

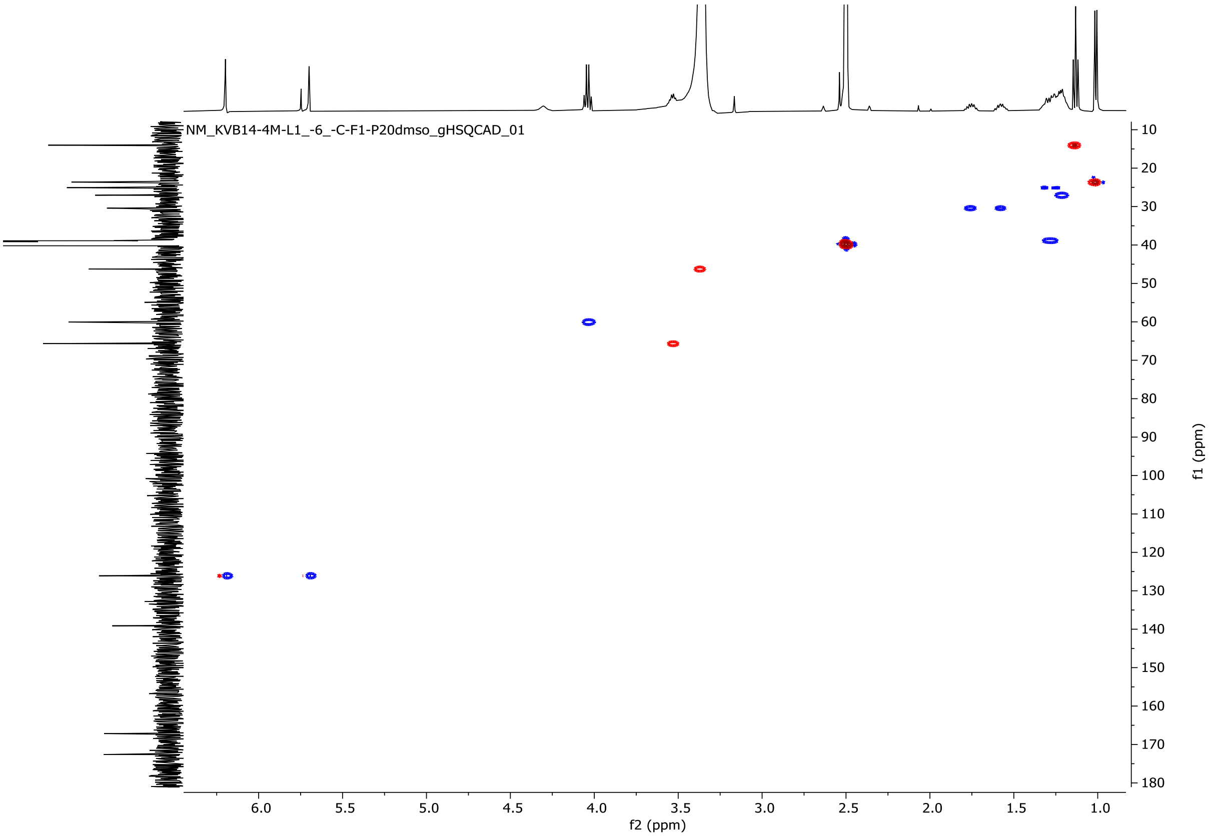

Figure S21. HSQC NMR spectrum of Compound **4** in DMSO-d_6 (600 MHz)

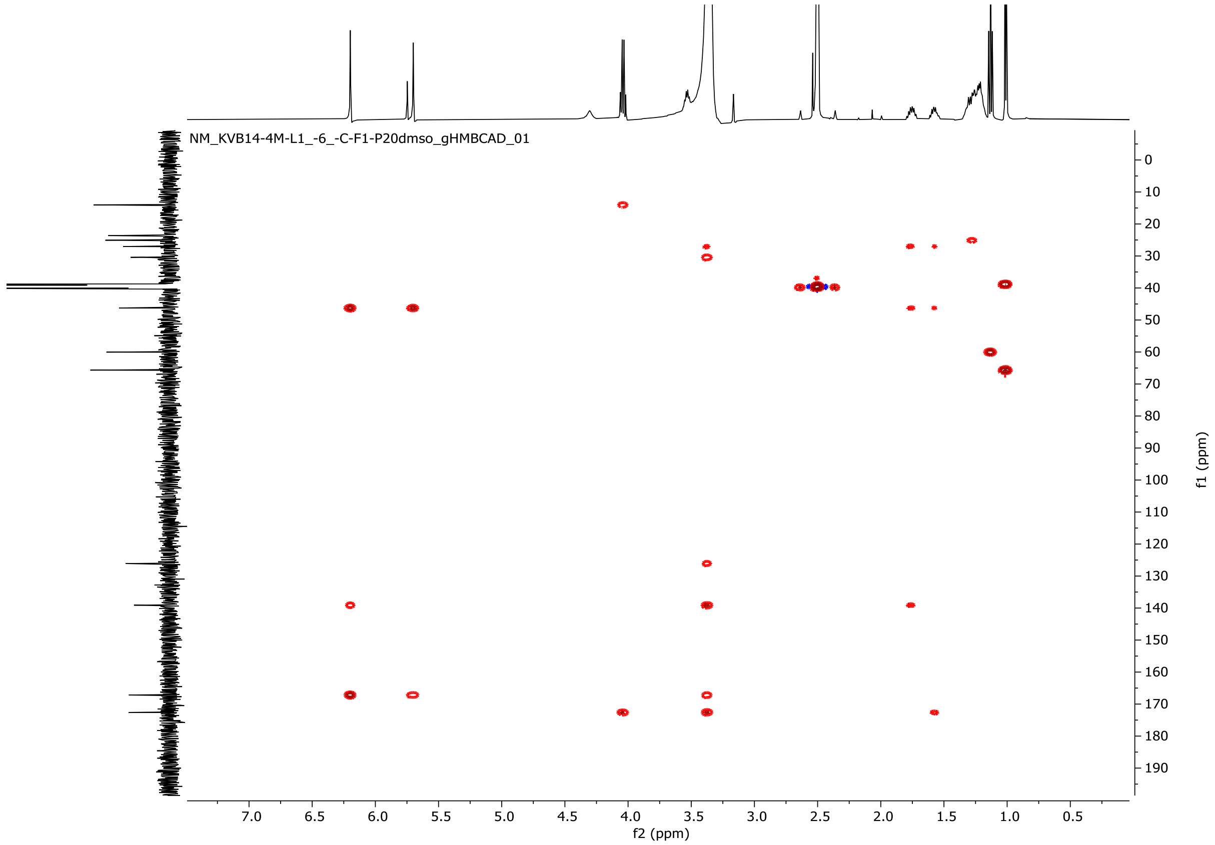

Figure S22. HMBC NMR spectrum of Compound **4** in DMSO-d_6 (600 MHz)
